# Supplementary material for: PRODH safeguards human naive pluripotency by limiting mitochondrial oxidative phosphorylation and reactive oxygen species production
Source: EMBO Rep. 2024 Mar 13;25(4):22. doi: 10.1038/s44319-024-00110-z (PMC11014864; doi:10.1038/s44319-024-00110-z)
Supplement: Supplementary file 1 — Appendix [file 44319_2024_110_MOESM1_ESM.pdf]

**Appendix for**

# PRODH safeguards human naïve pluripotency by limiting mitochondrial oxidative phosphorylation and reactive oxygen species production

Cheng Chen, Qianyu Liu, Wenjie Chen, Zhiyuan Gong, Bo Kang, Meihua Sui, Liming Huang, and Ying-Jie Wang

Table of contents:

Appendix Figure S1________________________Page 2

Appendix Figure S2________________________Page 3

Appendix Figure S3________________________Page 4

Appendix Figure S4________________________Page 5-6

Appendix Figure S5________________________Page 7

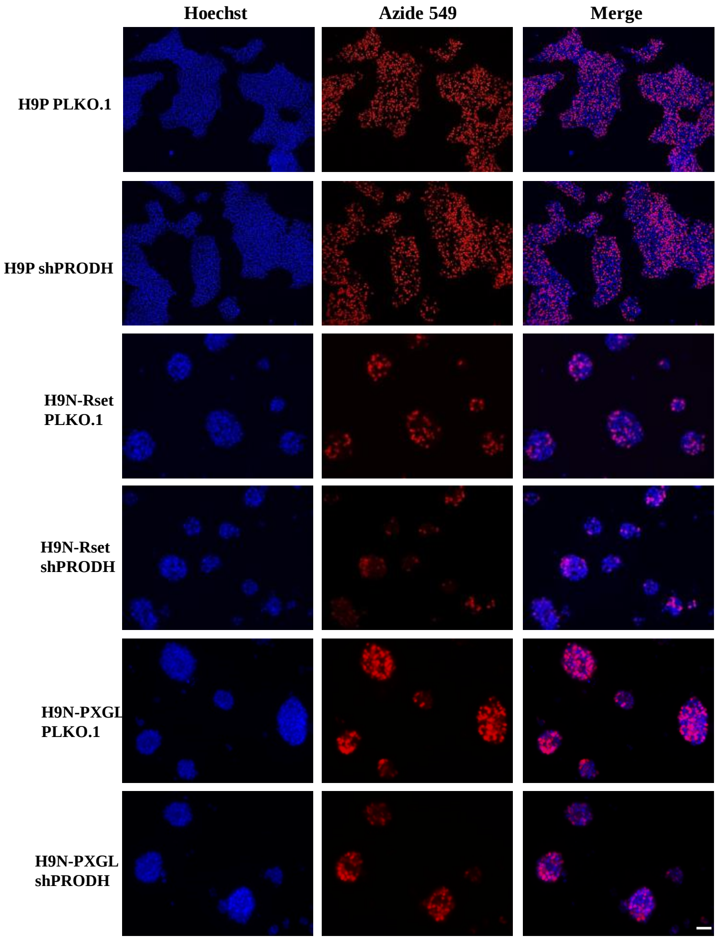


**Appendix Figure S1. The impact of PRODH on pluripotency in the naïve state is not attributed to its suppression of cellular proliferation.** Cell proliferation rate represented by EdU incorporation was assessed by an EdU assay. Nuclei were stained by Hoechst 33342. Scale bars, 50 μm. Representative images were presented.

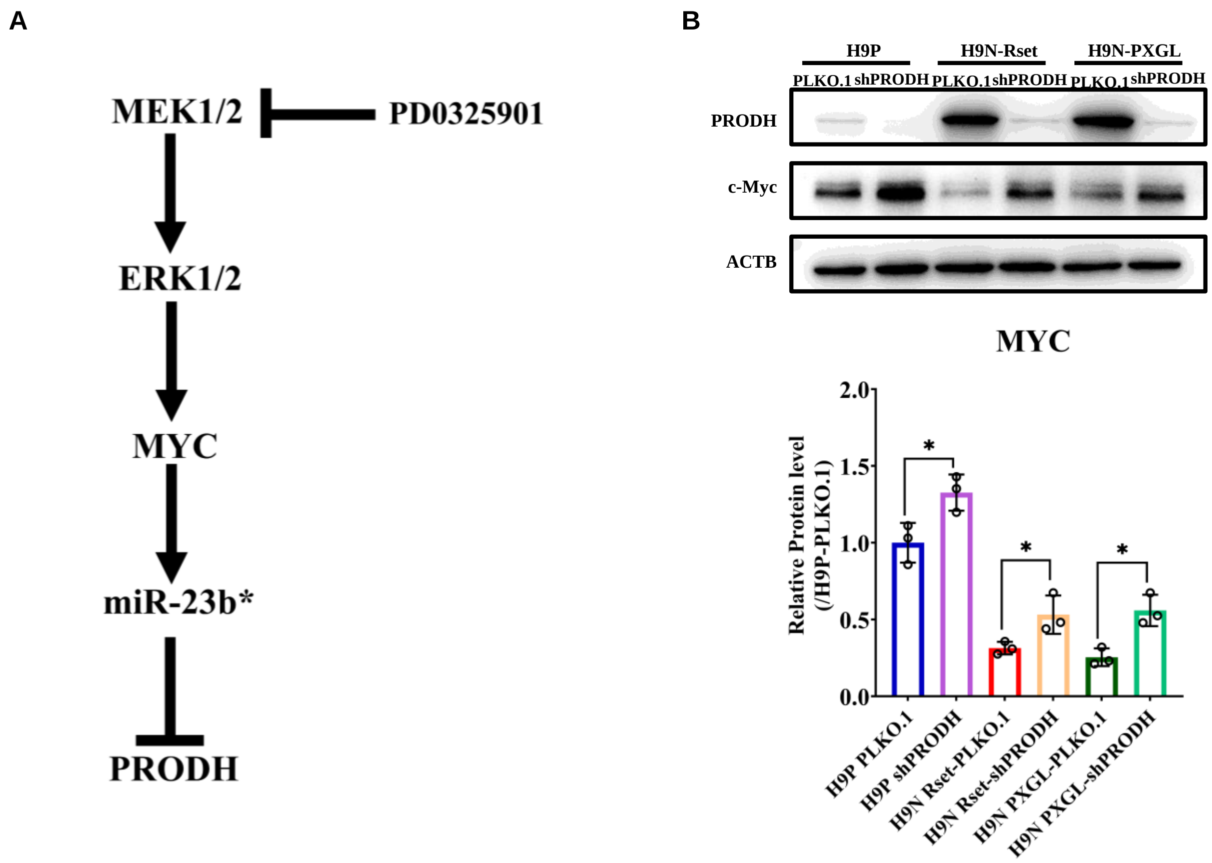


**Appendix Figure S2. MEK/ERK-MYC-PRODH signaling pathway plays a pivotal role in regulating PRODH expression between primed and naive states.**
(A) Summary of the MEK/ERK-MYC-PRODH signaling pathway when adding PD0325901.
(B) The protein levels of c-Myc in PLKO.1 and shPRODH hESCs. Quantifications of the WB bands relative to β-actin and normalized to H9P PLKO.1 values were presented. The statistical significance was analyzed using unpaired two-tailed Student's t-test. NS, not significant ($P > 0.05$). *$P < 0.05$, **$P < 0.01$, ***$P < 0.001$. The data shown were all from three independent biological replicates. Data were presented as mean ±SD.

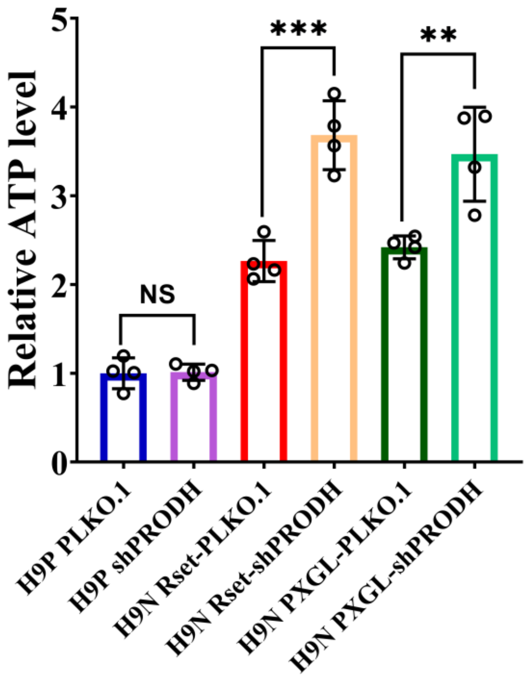


**Appendix Figure S3. The suppression of PRODH amplifies the generation of ATP in both Rset and PXGL hESCs.** For ATP level analysis, cells were first lysed. After centrifugation, the supernatant was prepared for intracellular ATP level measurement using a firefly luciferase-based assay. Quantitation was performed and normalized to H9P PLKO.1 values. The statistical significance was analyzed using unpaired two-tailed Student's t-test. NS, not significant ($P > 0.05$). * $P < 0.05$, ** $P < 0.01$, *** $P < 0.001$. The data shown were all from four technical replicates. Data were presented as mean ±SD.

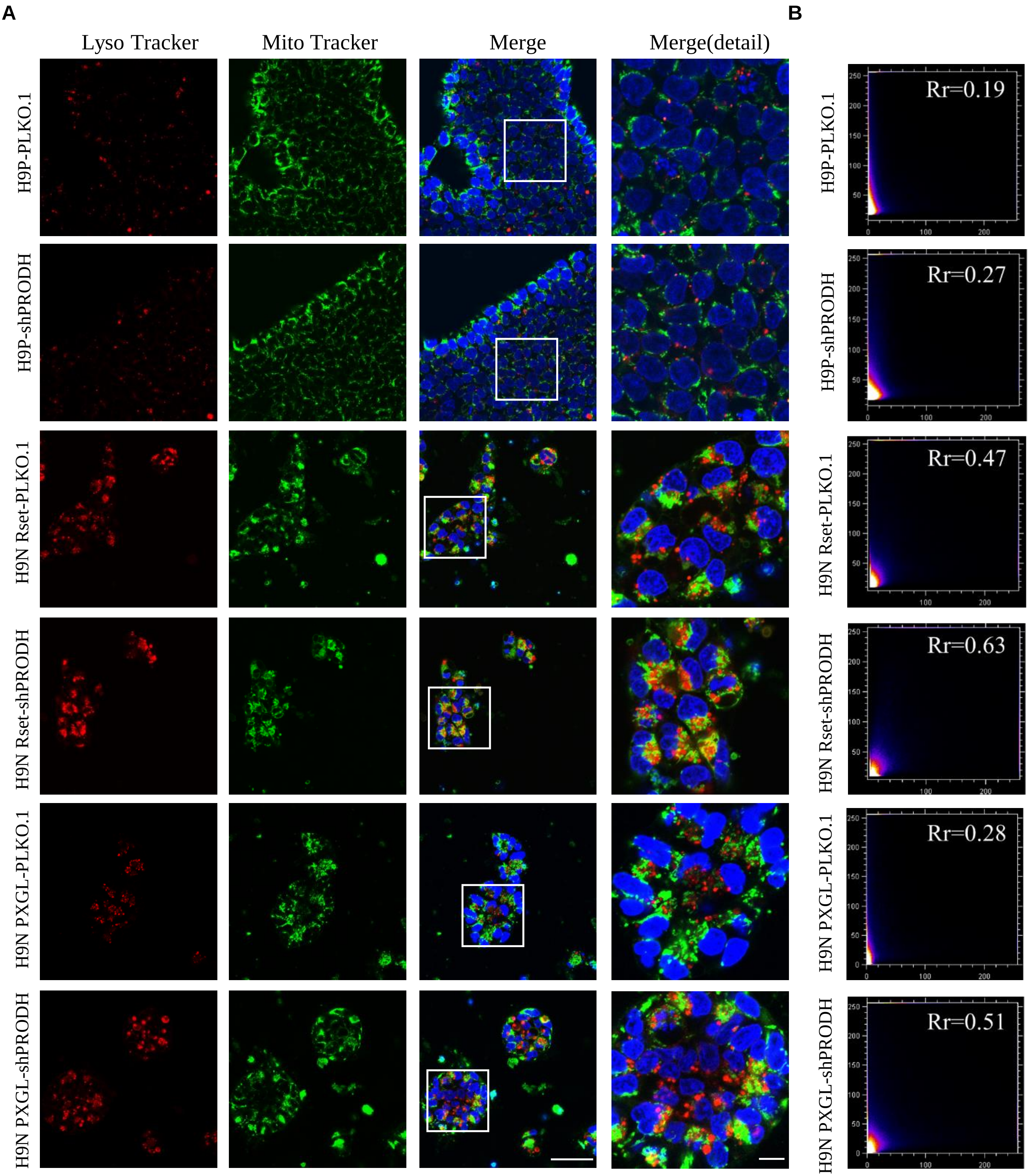

A
B
Lyso Tracker
Mito Tracker
Merge
Merge(detail)
H9P-PLKO.1
H9P-shPRODH
H9N Rset-PLKO.1
H9N Rset-shPRODH
H9N PXGL-PLKO.1
H9N PXGL-shPRODH
Rr=0.19
Rr=0.27
Rr=0.47
Rr=0.63
Rr=0.28
Rr=0.51

**Appendix Figure S4. The inhibition of PRODH facilitates the co-localization of mitochondria and lysosomes.** Mitochondria in both groups were stained with MitoTracker Green and LysoTracker Deep Red for 30 min at 37°C and visualized by confocal microscopy. Representative confocal micrographs were shown (A). Scale bars, 50 μm (left), 10 μm (right). The co-localization of mitochondria and lysosomes was analyzed using the ImageJ software Colocalization Finder (B). Data were obtained from three separate experiments, and more than 10 cells were analyzed in each experiment.

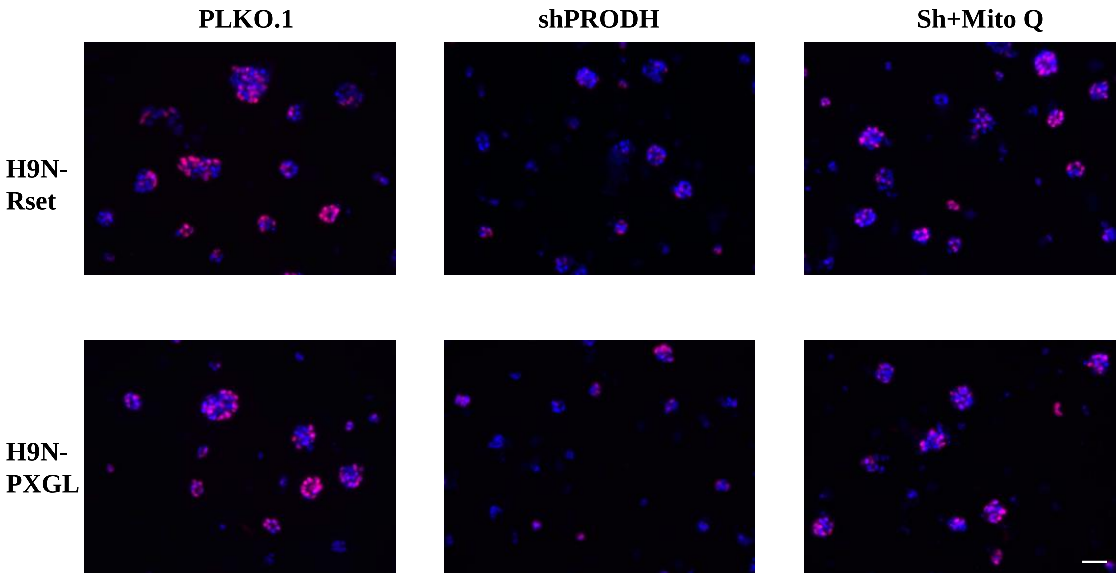


**Appendix Figure S5. Reduction of ROS restores the proliferative capacity of Rset-shPRODH and PXGL-shPRODH cells.** Rset-shPRODH and PXGL-shPRODH cells were treated with 50 nM MitoQ for 3 hours, and cultured for an additional 12 hours. Cell proliferation rate represented by EdU incorporation was assessed by an EdU assay. Representative images were shown.Scale bars, 50 µM.
