# Supplementary figures and images for: PRODH safeguards human naive pluripotency by limiting mitochondrial oxidative phosphorylation and reactive oxygen species production
Source: EMBO Rep. 2024 Mar 13;25(4):22. doi: 10.1038/s44319-024-00110-z (PMC11014864; doi:10.1038/s44319-024-00110-z)

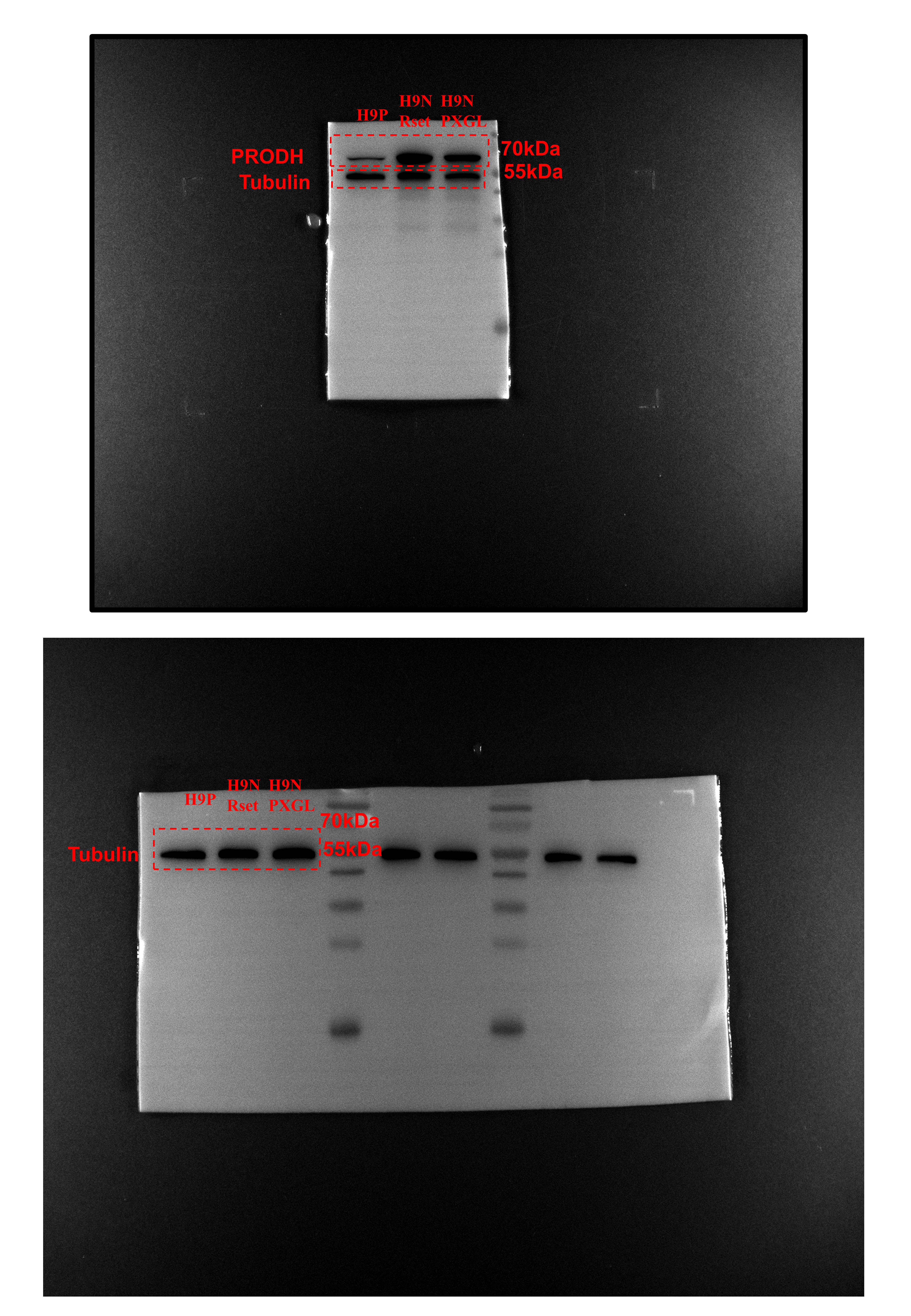

Supplement: Supplementary file 5 — Source Data Fig. 1 [file 44319_2024_110_MOESM5_ESM.zip › Figure1/1C/western-1C.TIF]

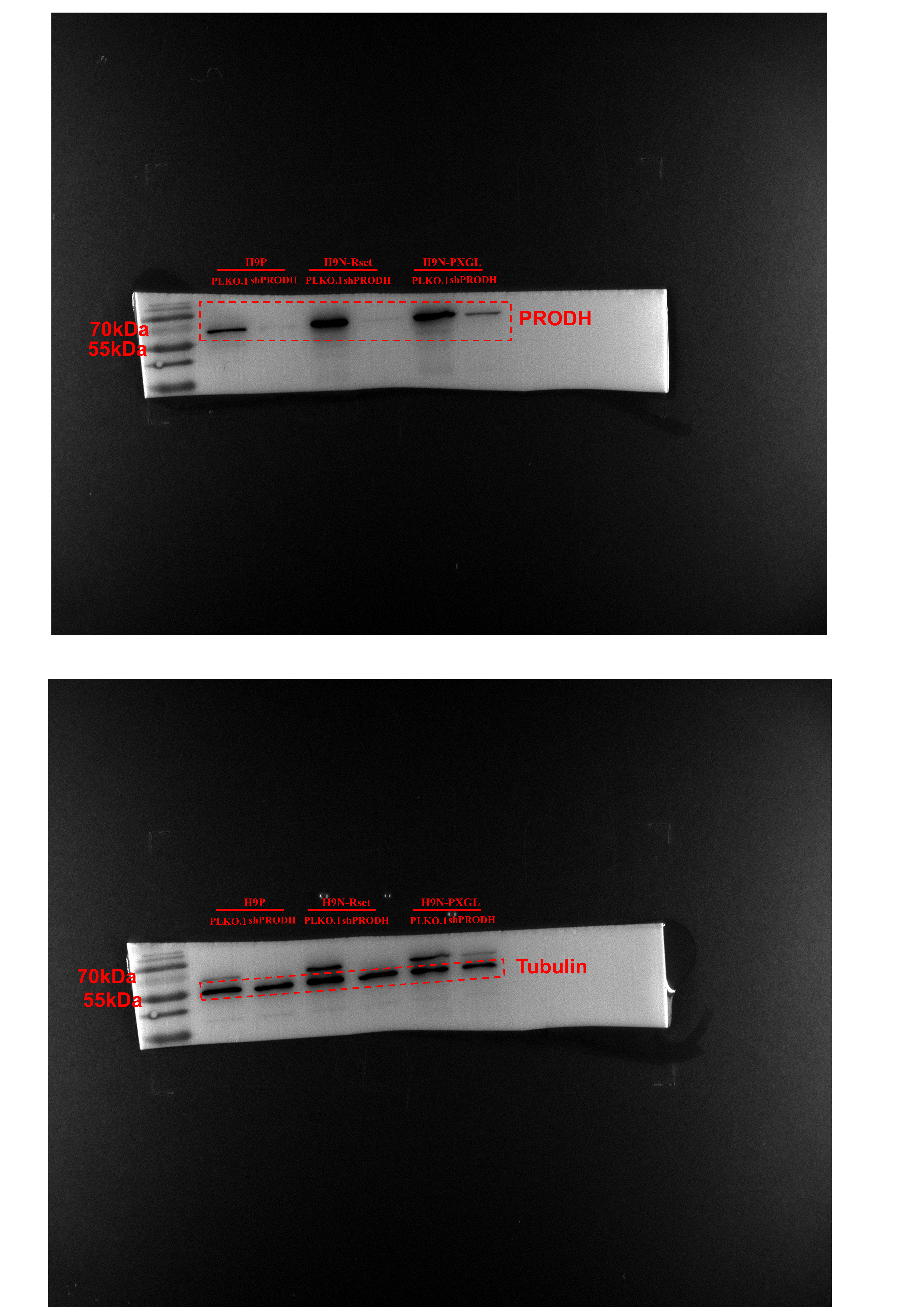

Supplement: Supplementary file 5 — Source Data Fig. 1 [file 44319_2024_110_MOESM5_ESM.zip › Figure1/1D/western-1D.tif]

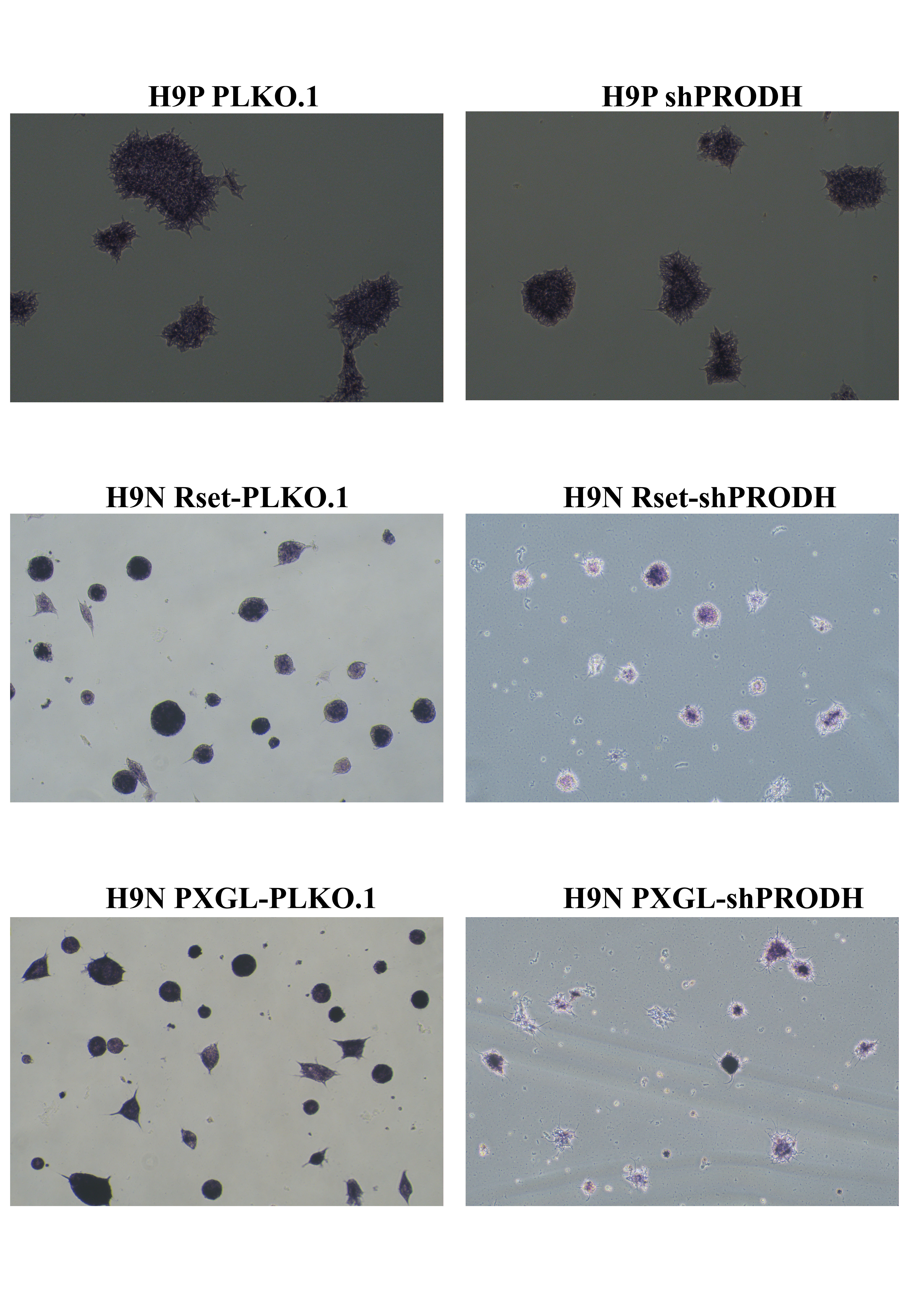

Supplement: Supplementary file 5 — Source Data Fig. 1 [file 44319_2024_110_MOESM5_ESM.zip › Figure1/1E/AP stain-1E.TIF]

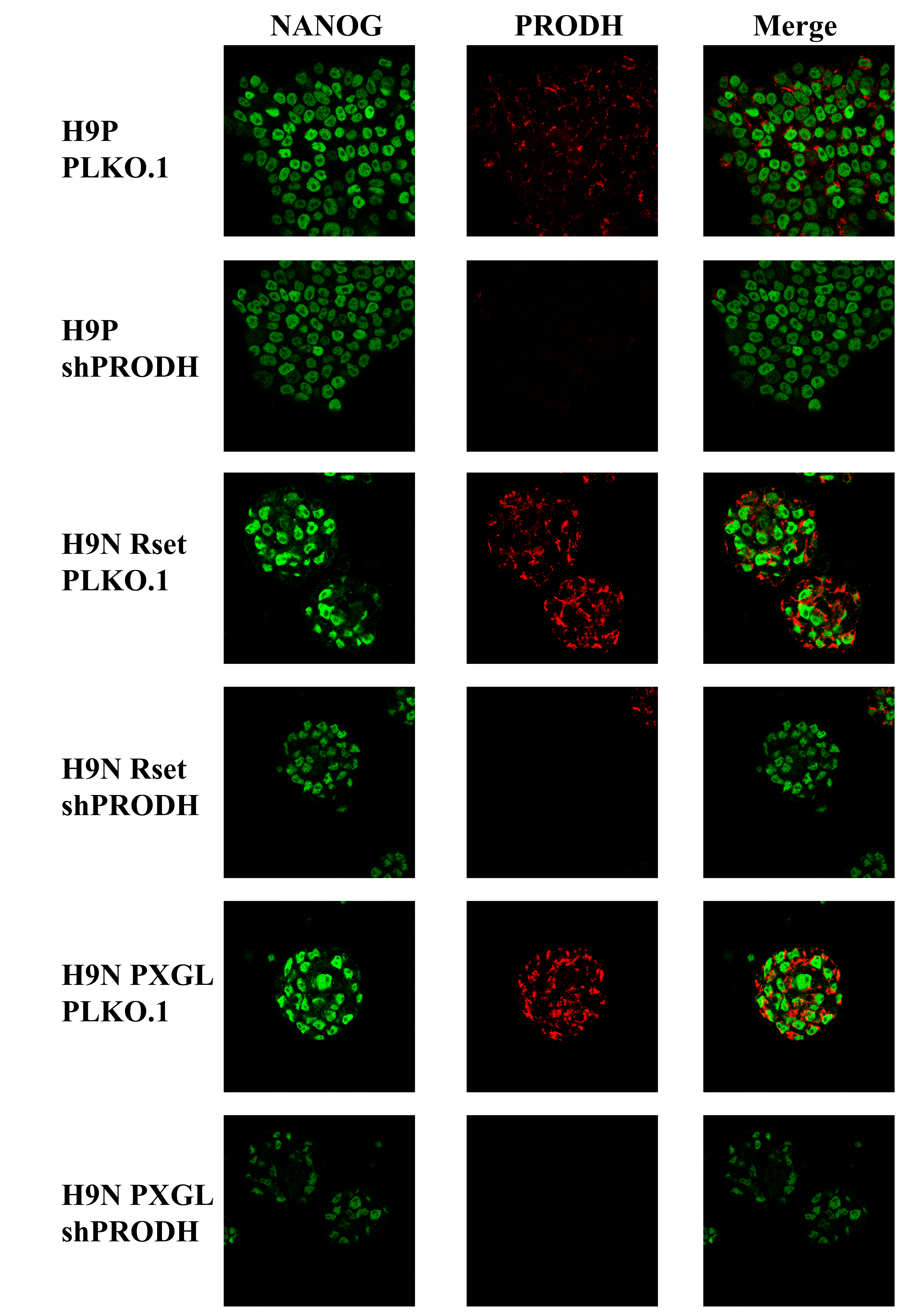

Supplement: Supplementary file 5 — Source Data Fig. 1 [file 44319_2024_110_MOESM5_ESM.zip › Figure1/1F/IF-1F.tif]

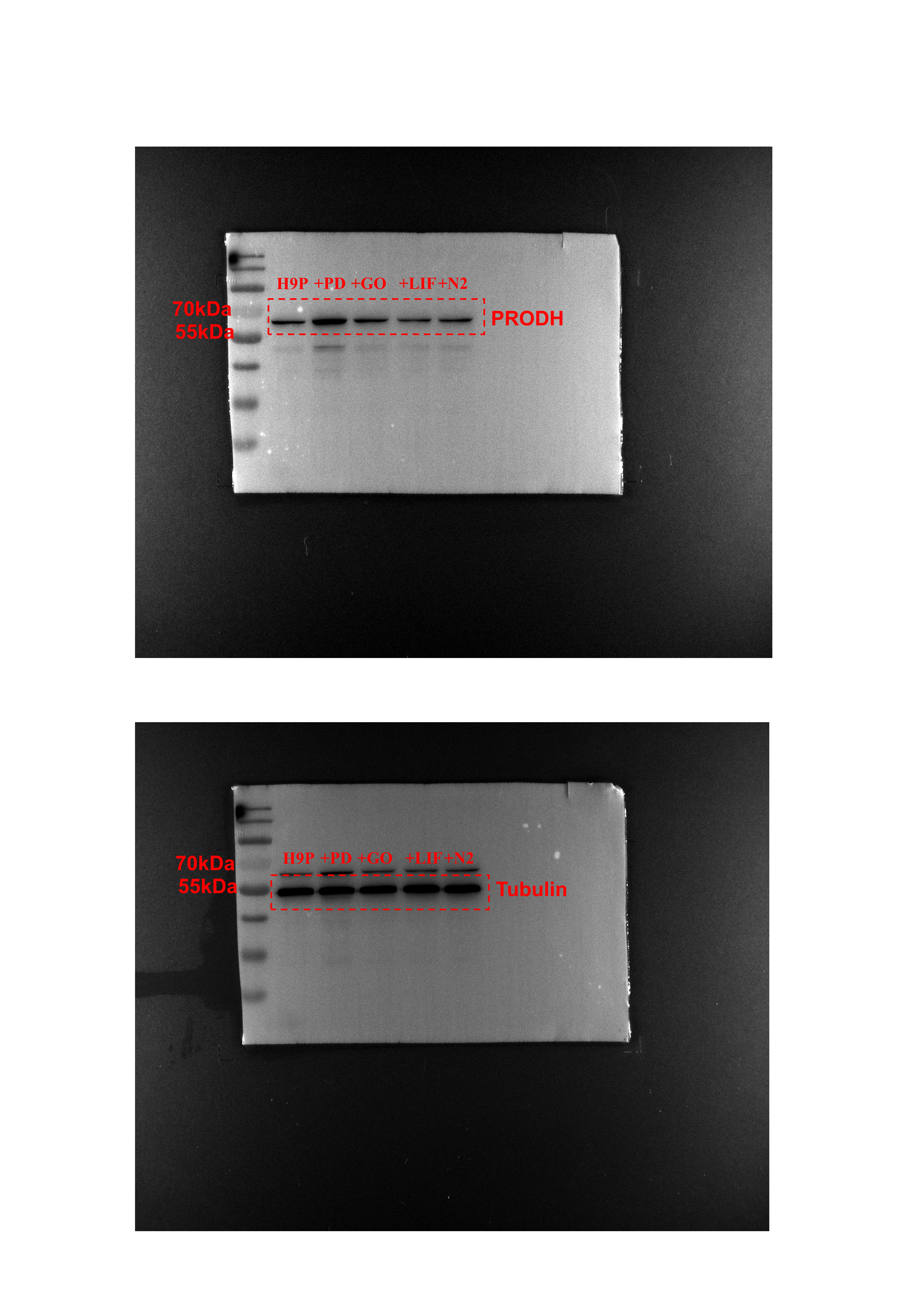

Supplement: Supplementary file 6 — Source Data Fig. 2 [file 44319_2024_110_MOESM6_ESM.zip › Figure2/2C/western-2C.tif]

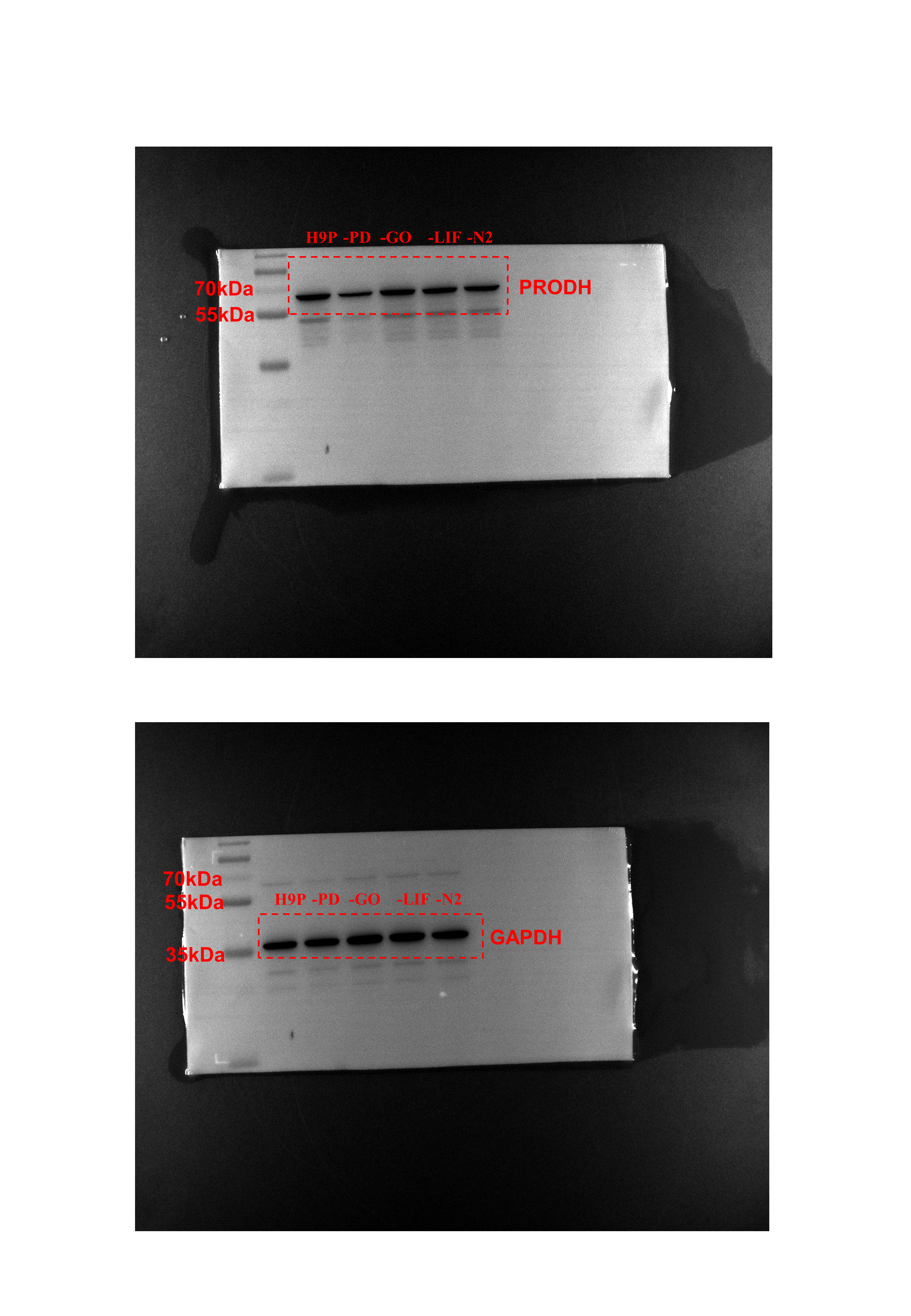

Supplement: Supplementary file 6 — Source Data Fig. 2 [file 44319_2024_110_MOESM6_ESM.zip › Figure2/2E/western-2E.tif]

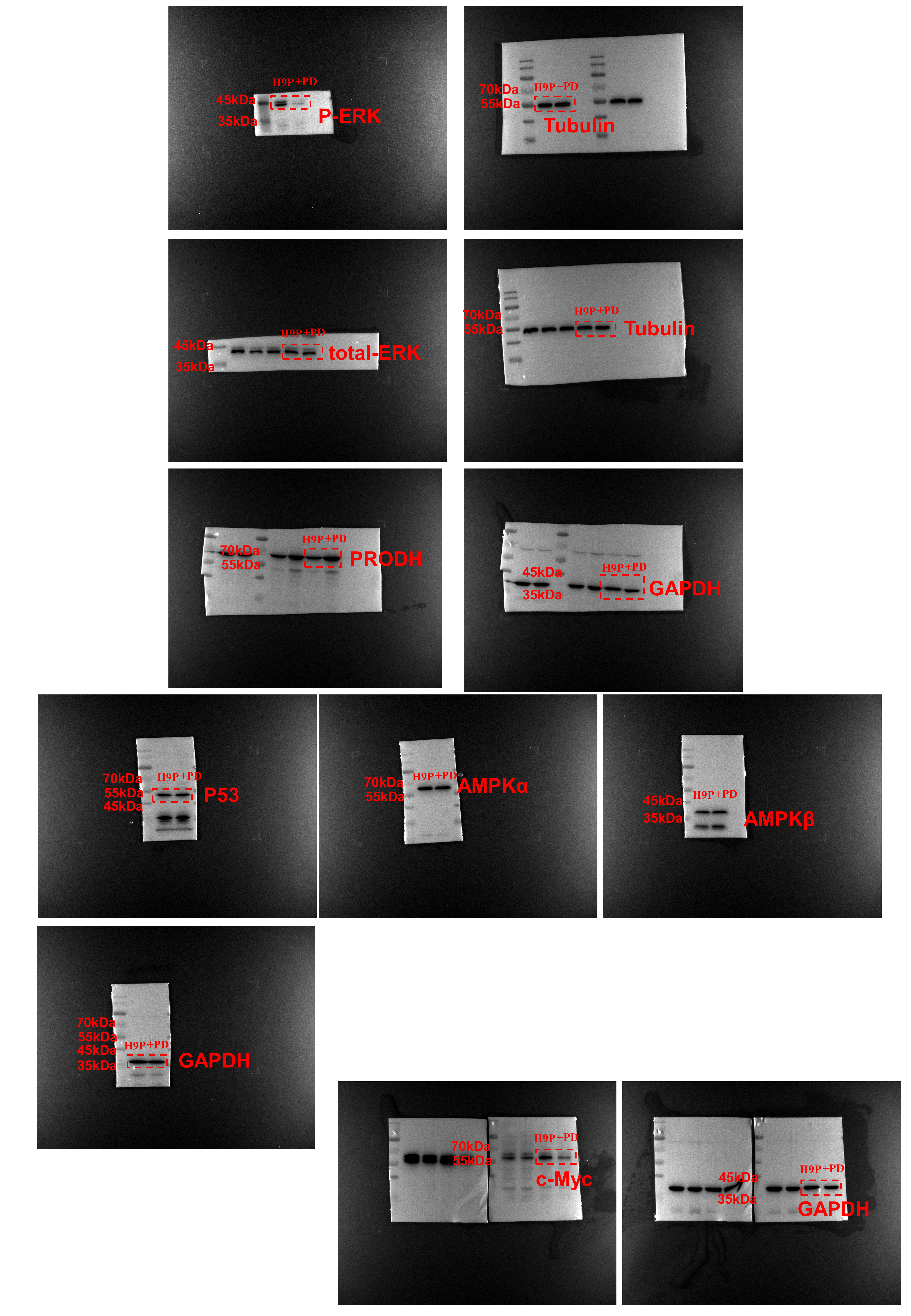

Supplement: Supplementary file 6 — Source Data Fig. 2 [file 44319_2024_110_MOESM6_ESM.zip › Figure2/2F/western-2F.tif]

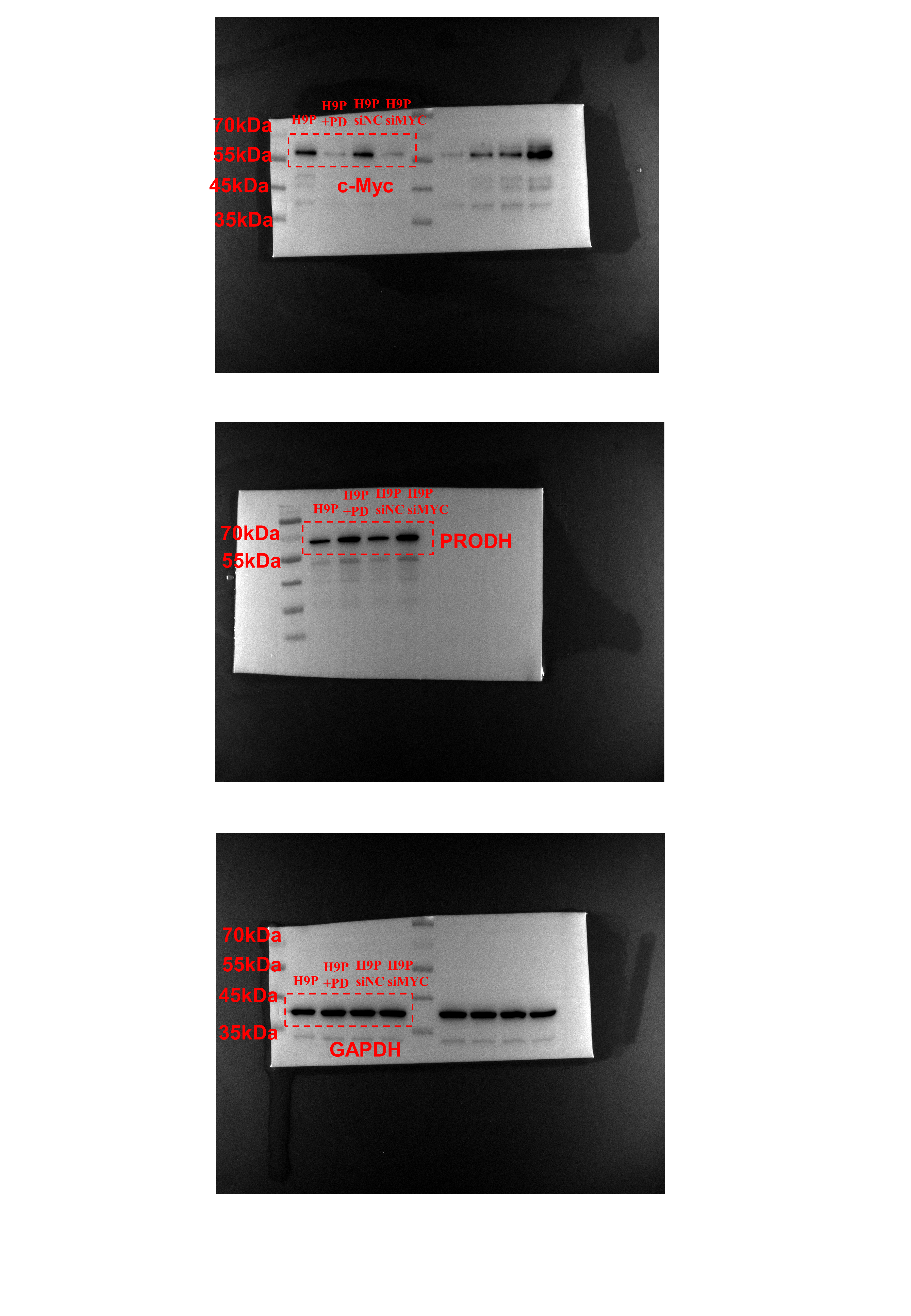

Supplement: Supplementary file 6 — Source Data Fig. 2 [file 44319_2024_110_MOESM6_ESM.zip › Figure2/2H/western-2H.tif]

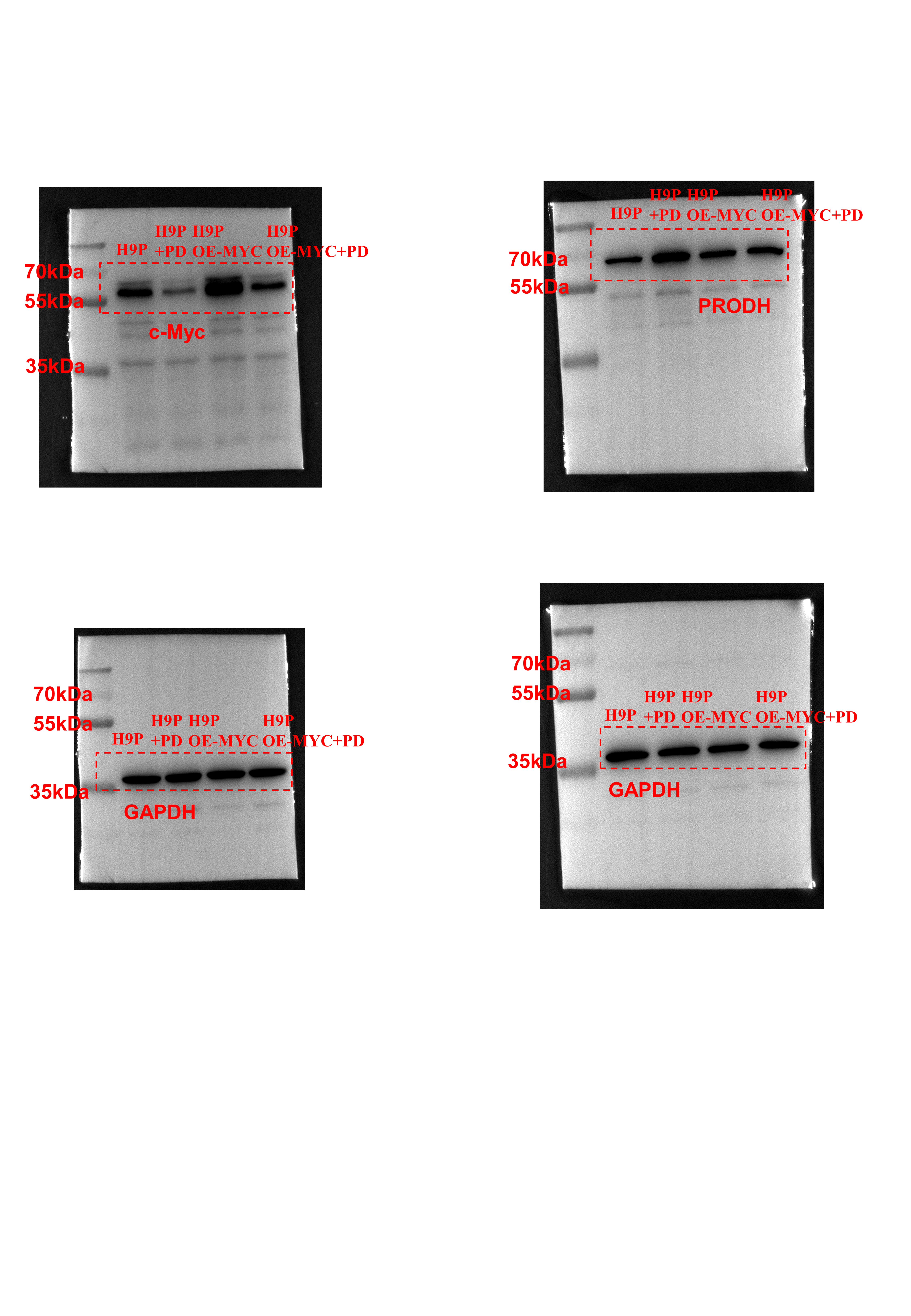

Supplement: Supplementary file 6 — Source Data Fig. 2 [file 44319_2024_110_MOESM6_ESM.zip › Figure2/2J/western-2J.tif]

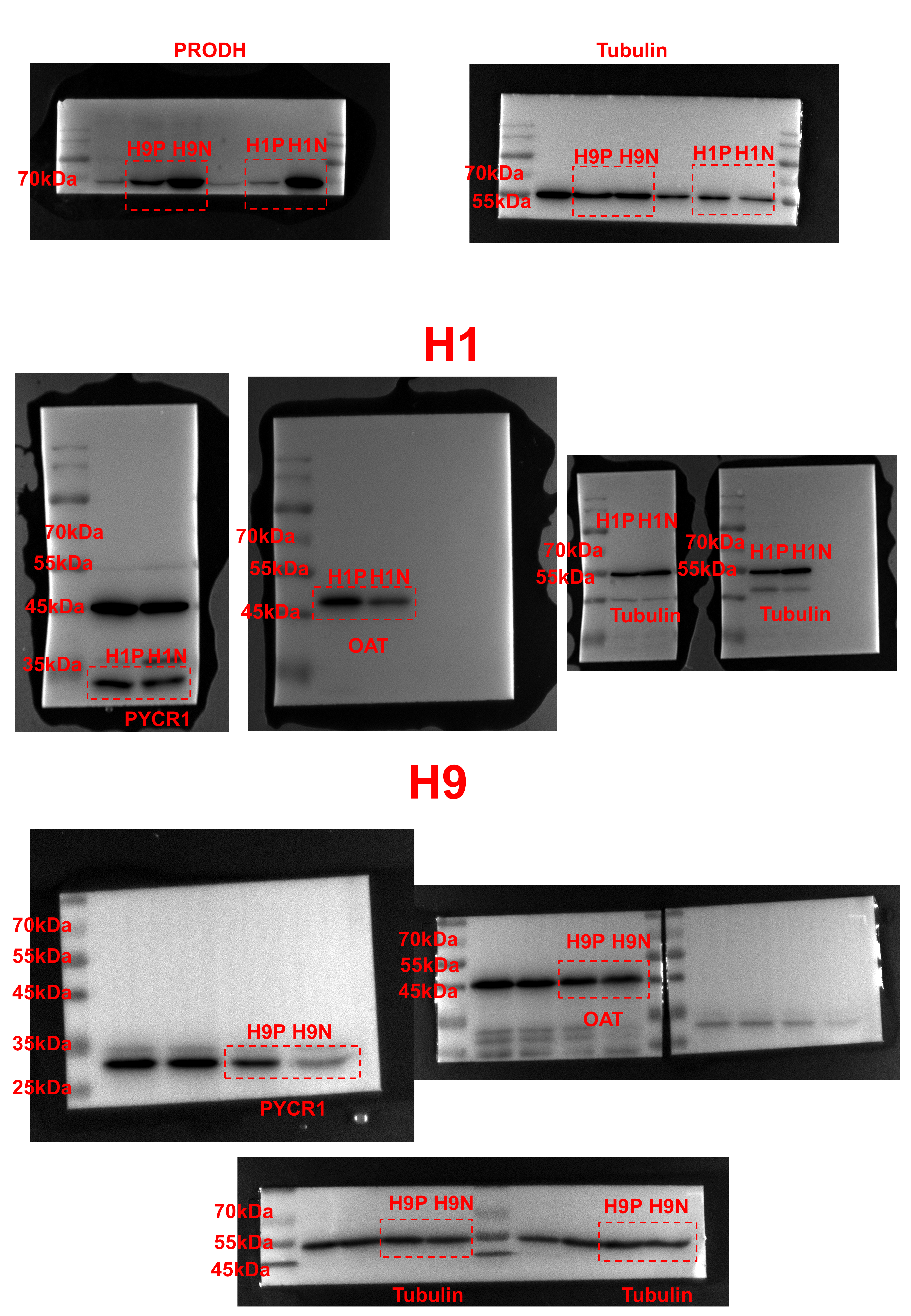

Supplement: Supplementary file 7 — Source Data Fig. 3 [file 44319_2024_110_MOESM7_ESM.zip › Figure3/3B/western-3B.tif]

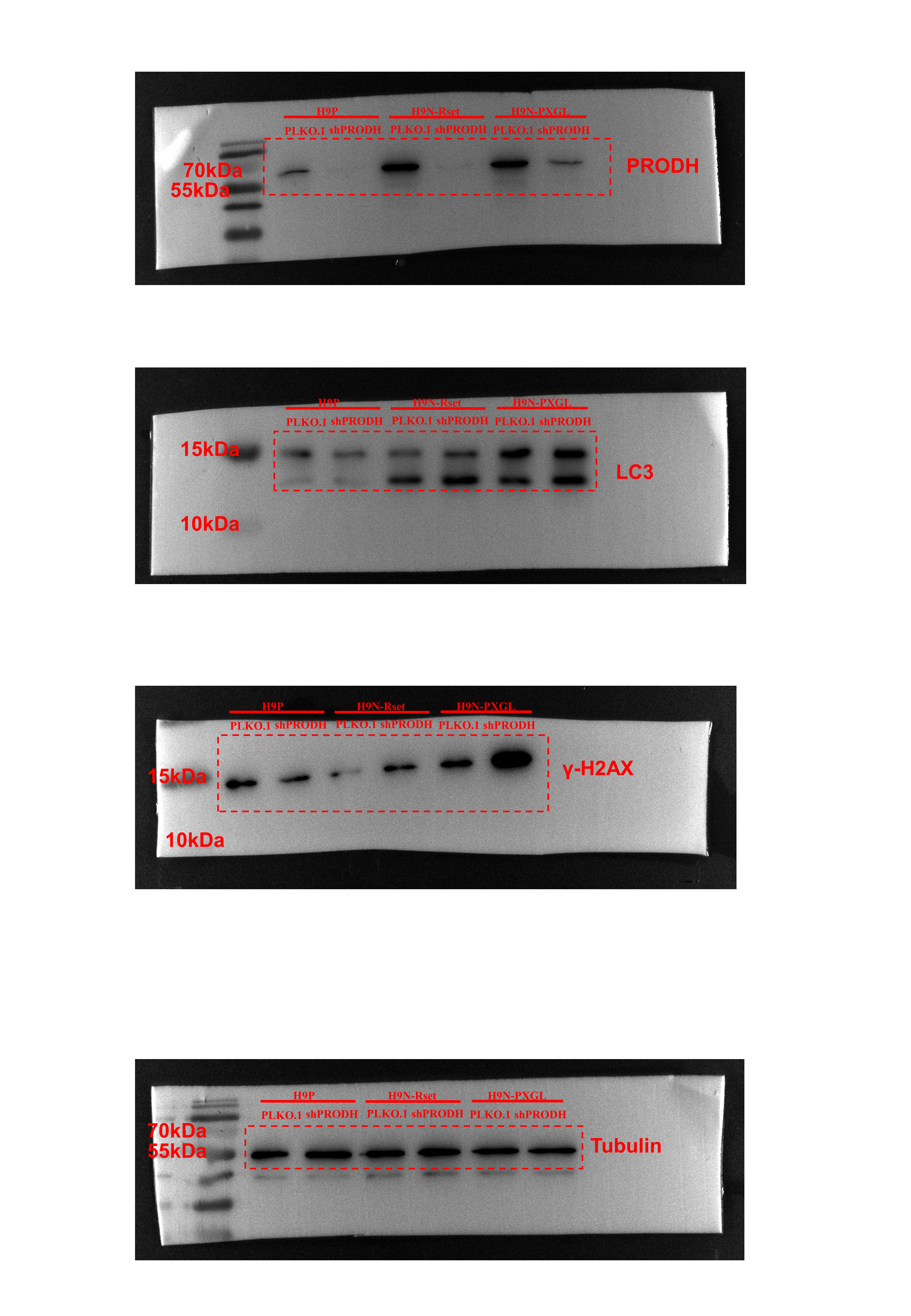

Supplement: Supplementary file 9 — Source Data Fig. 5 [file 44319_2024_110_MOESM9_ESM.zip › Figure5/5B/western-5B.tif]

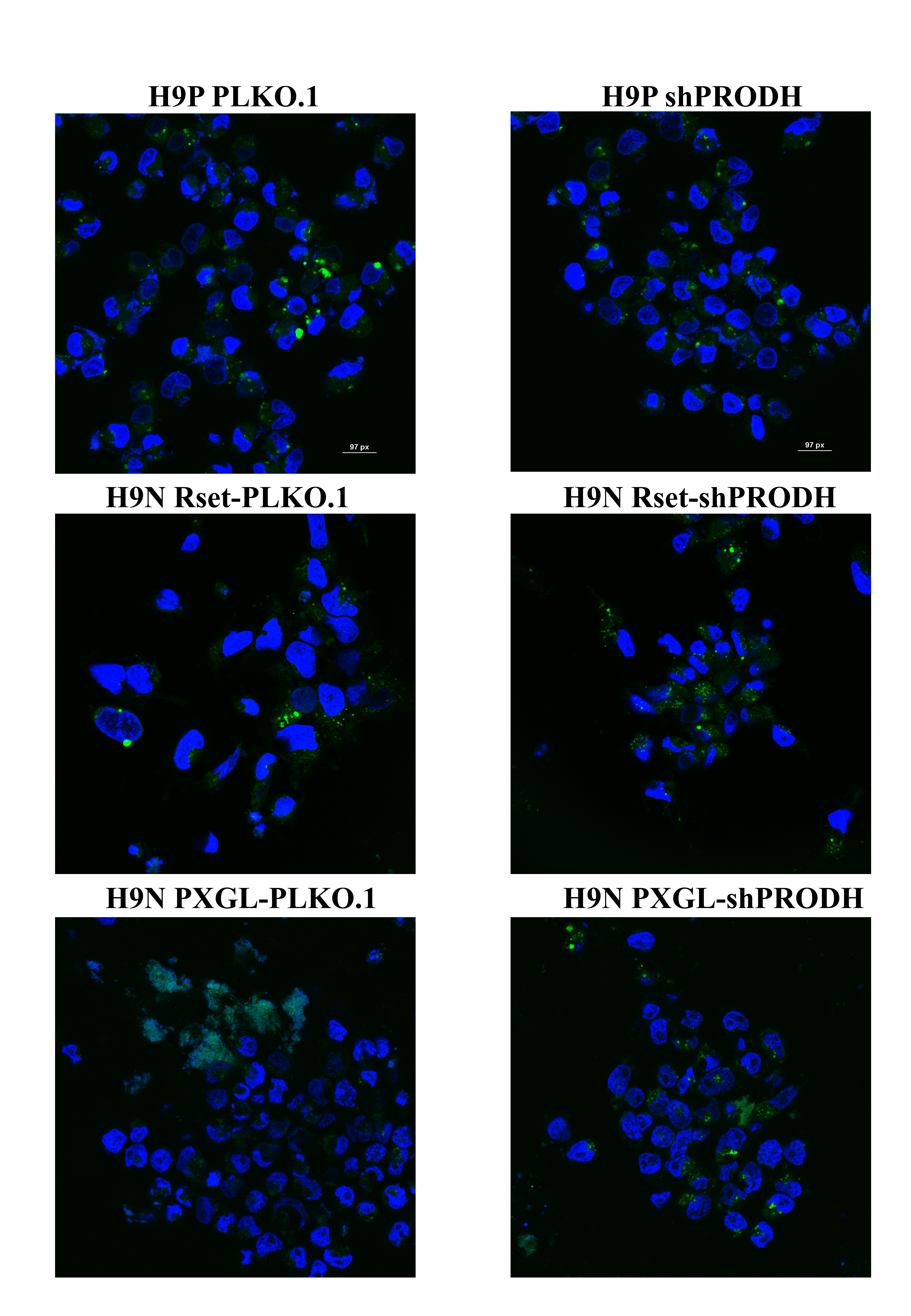

Supplement: Supplementary file 9 — Source Data Fig. 5 [file 44319_2024_110_MOESM9_ESM.zip › Figure5/5D/CYTO ID-Confocol image.tif]

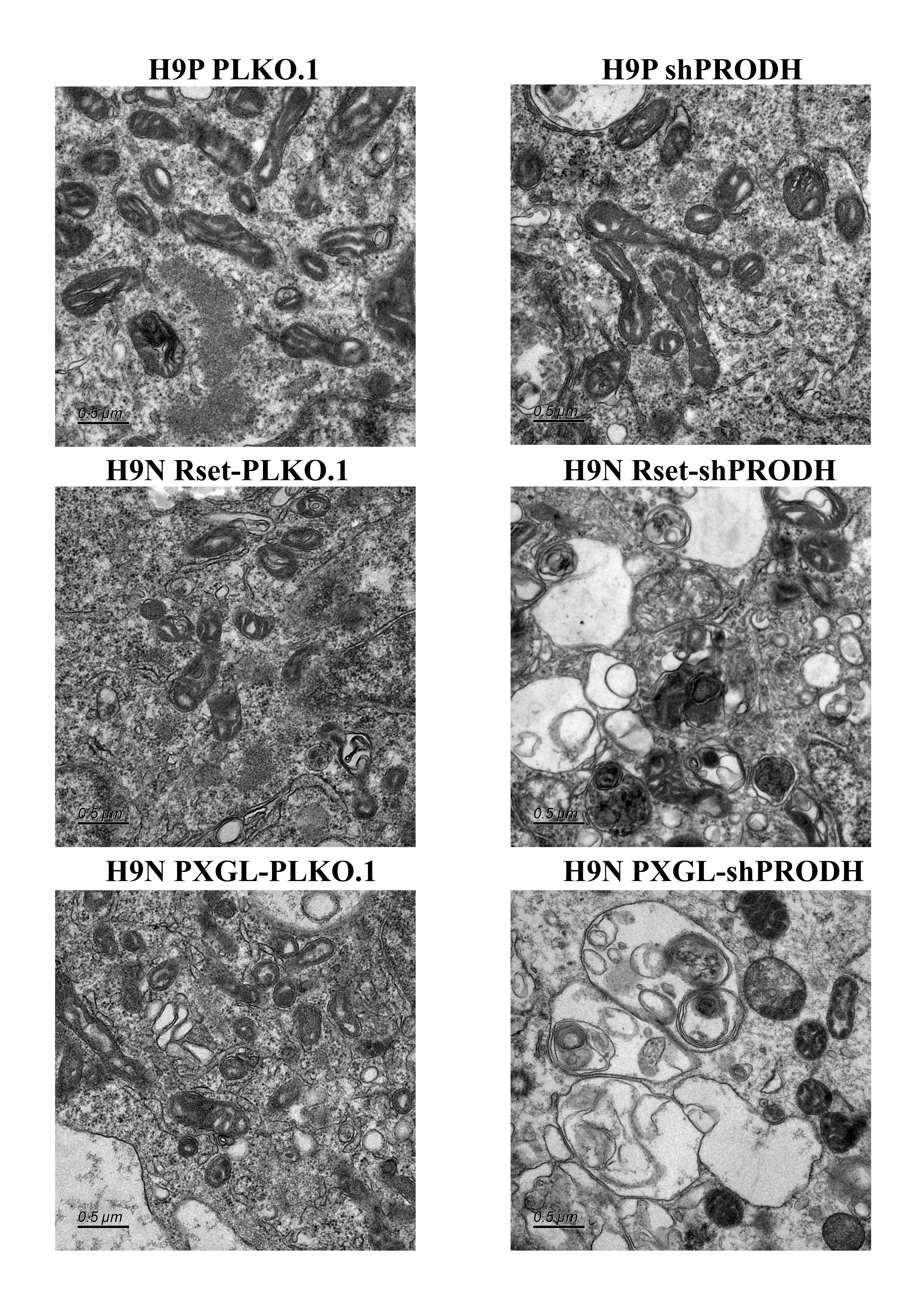

Supplement: Supplementary file 9 — Source Data Fig. 5 [file 44319_2024_110_MOESM9_ESM.zip › Figure5/5E/TEM-hESC mitophagosome.tif]

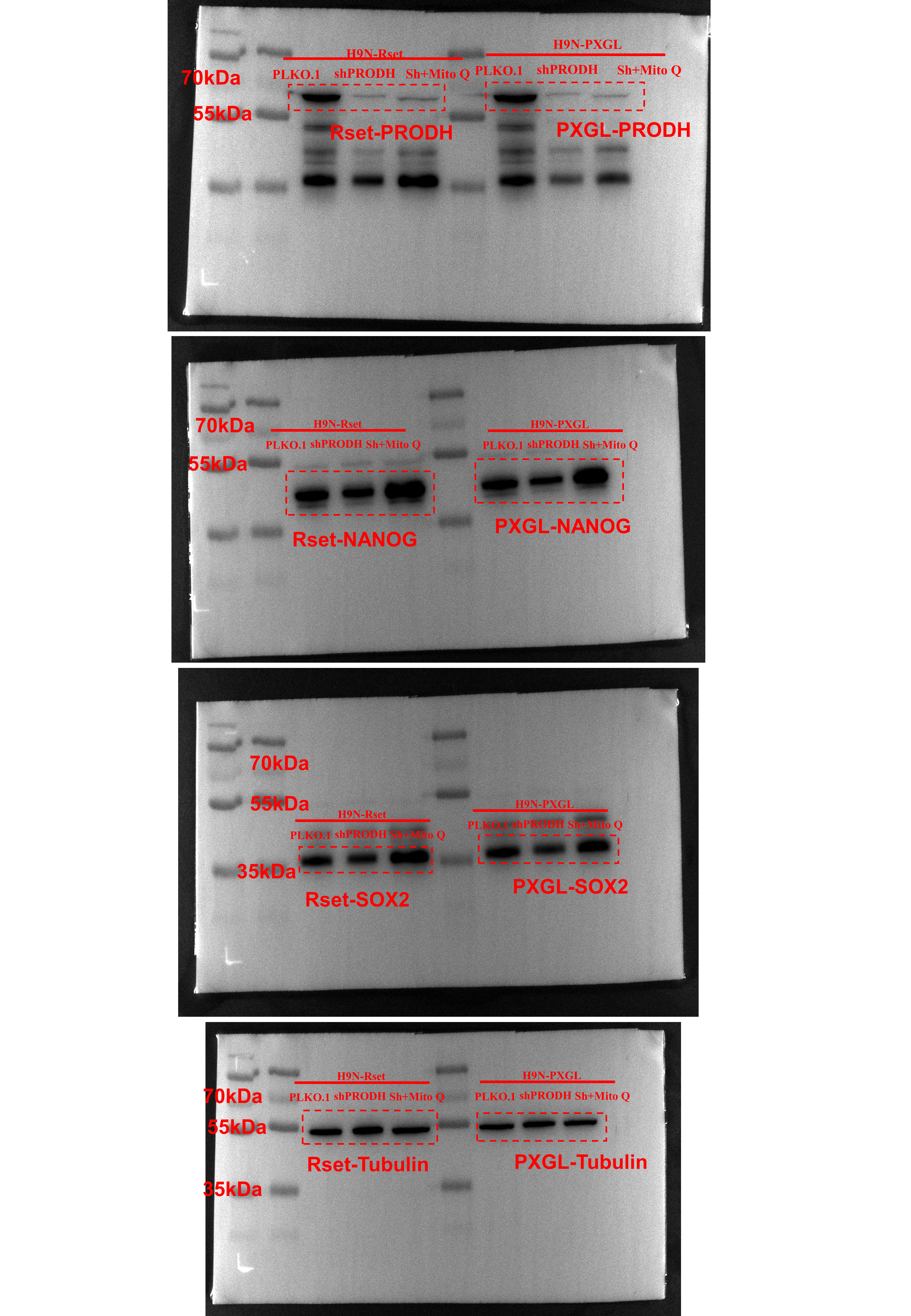

Supplement: Supplementary file 9 — Source Data Fig. 5 [file 44319_2024_110_MOESM9_ESM.zip › Figure5/5F/western-5F.tif]

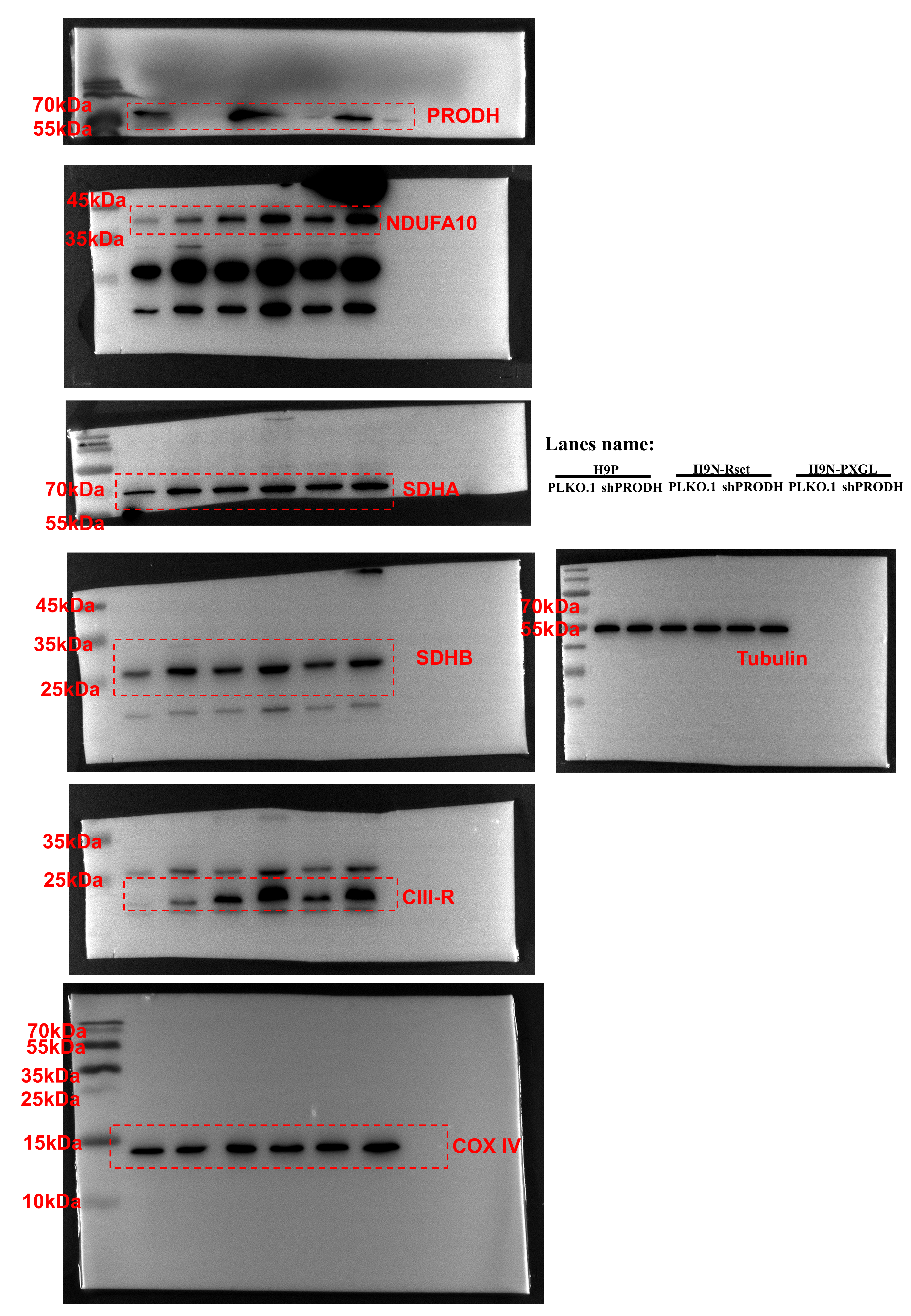

Supplement: Supplementary file 10 — Source Data Fig. 6 [file 44319_2024_110_MOESM10_ESM.zip › Figure6/6B/western-6B.tif]

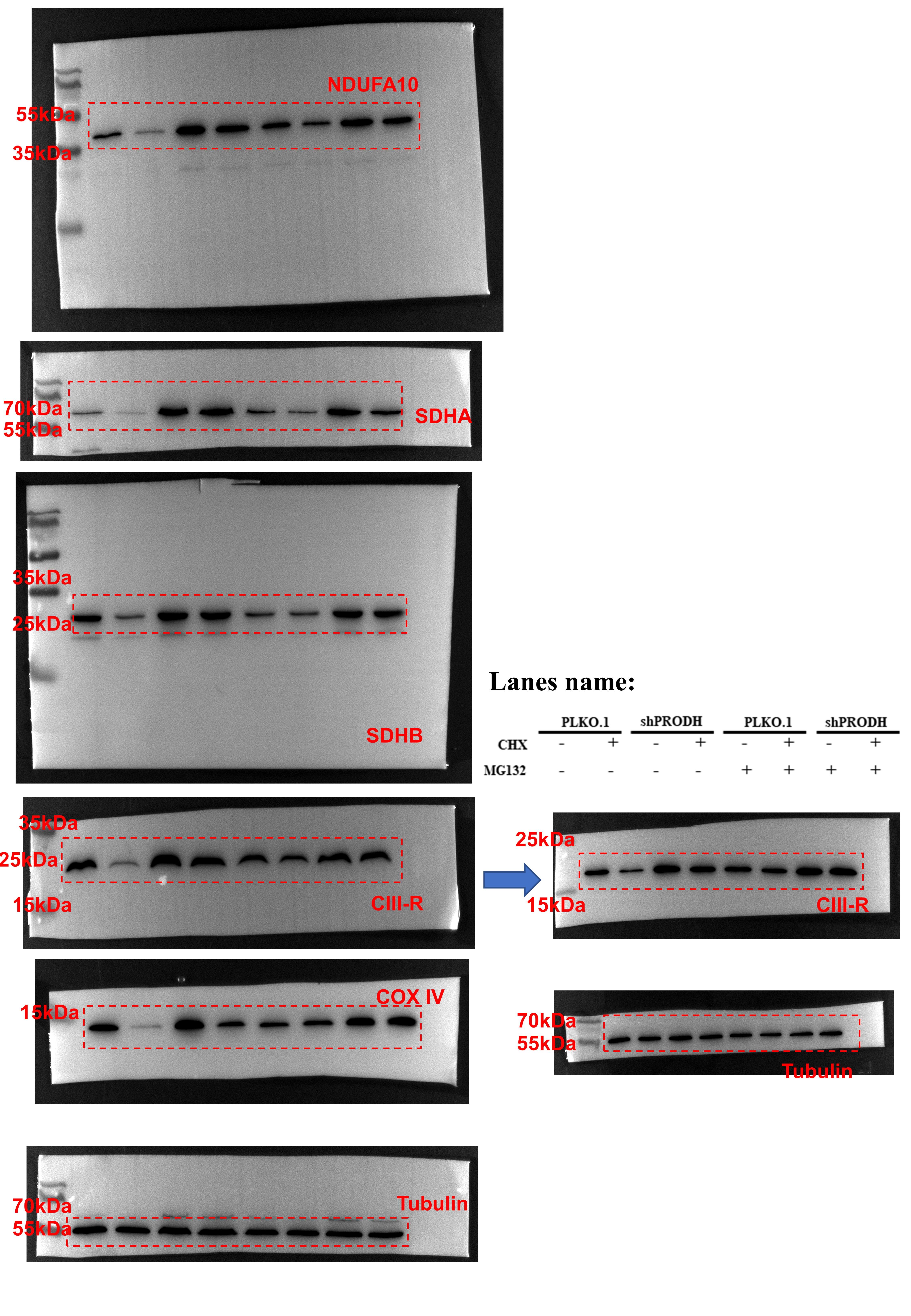

Supplement: Supplementary file 10 — Source Data Fig. 6 [file 44319_2024_110_MOESM10_ESM.zip › Figure6/6F/western-6E.tif]

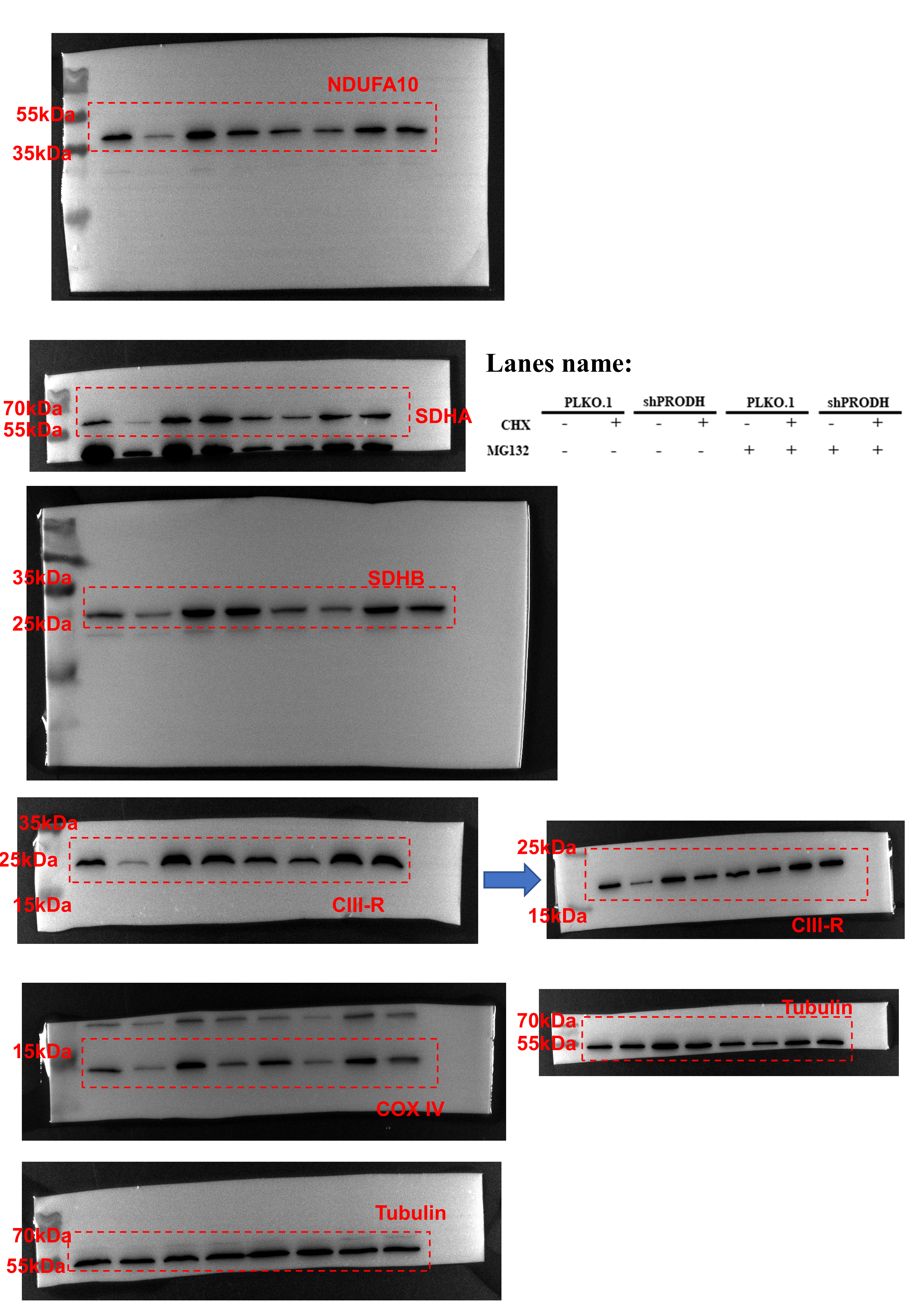

Supplement: Supplementary file 10 — Source Data Fig. 6 [file 44319_2024_110_MOESM10_ESM.zip › Figure6/6G/western-6F.tif]
