# Supplementary material for: PRODH safeguards human naive pluripotency by limiting mitochondrial oxidative phosphorylation and reactive oxygen species production
Source: EMBO Rep. 2024 Mar 13;25(4):22. doi: 10.1038/s44319-024-00110-z (PMC11014864; doi:10.1038/s44319-024-00110-z)
Supplement: Supplementary file 12 — Expanded View Figures [file 44319_2024_110_MOESM12_ESM.pdf]

Expanded View Figures

Figure EV1. PRODH knockdown had little effect on primed hESCs.

(**A**) Alkaline phosphatase staining of primed and naive (Rset and PXGL) hESCs after PRODH KD. Images show the AP+ colony number in randomly selected fields. Scale bars, 2.5 mm. (**B**) The protein levels of the key pluripotency factors OCT4, NANOG, and SOX2 in PLKO.1 and shPRODH hESCs were examined by WB. (**C**) Quantifications of the WB bands relative to α-tubulin and normalized to H9P PLKO.1 values were shown. (**D**) Rset- and PXGL-induced naive H9 hESCs were harvested. QRT-PCR determined the mRNA levels of pluripotency marker and naive pluripotency marker genes. (**E**) There was no significant change in pluripotency marker and primed pluripotency marker genes between H9P-PLKO.1 and H9P-shPRODH hESCs. (**F**) PRODH KD in Rset and PXGL naive H9 cells resulted in increased transcription of mesendoderm markers. Data information: The statistical significance was analyzed using unpaired two-tailed Student's t-test in panels. NS, not significant ($P > 0.05$). *$P < 0.05$, **$P < 0.01$, ***$P < 0.001$. The data shown were all from three independent biological replicates. Each data point represents an independent biological replicate. Data were presented as mean ± SD.

 © The Author(s)

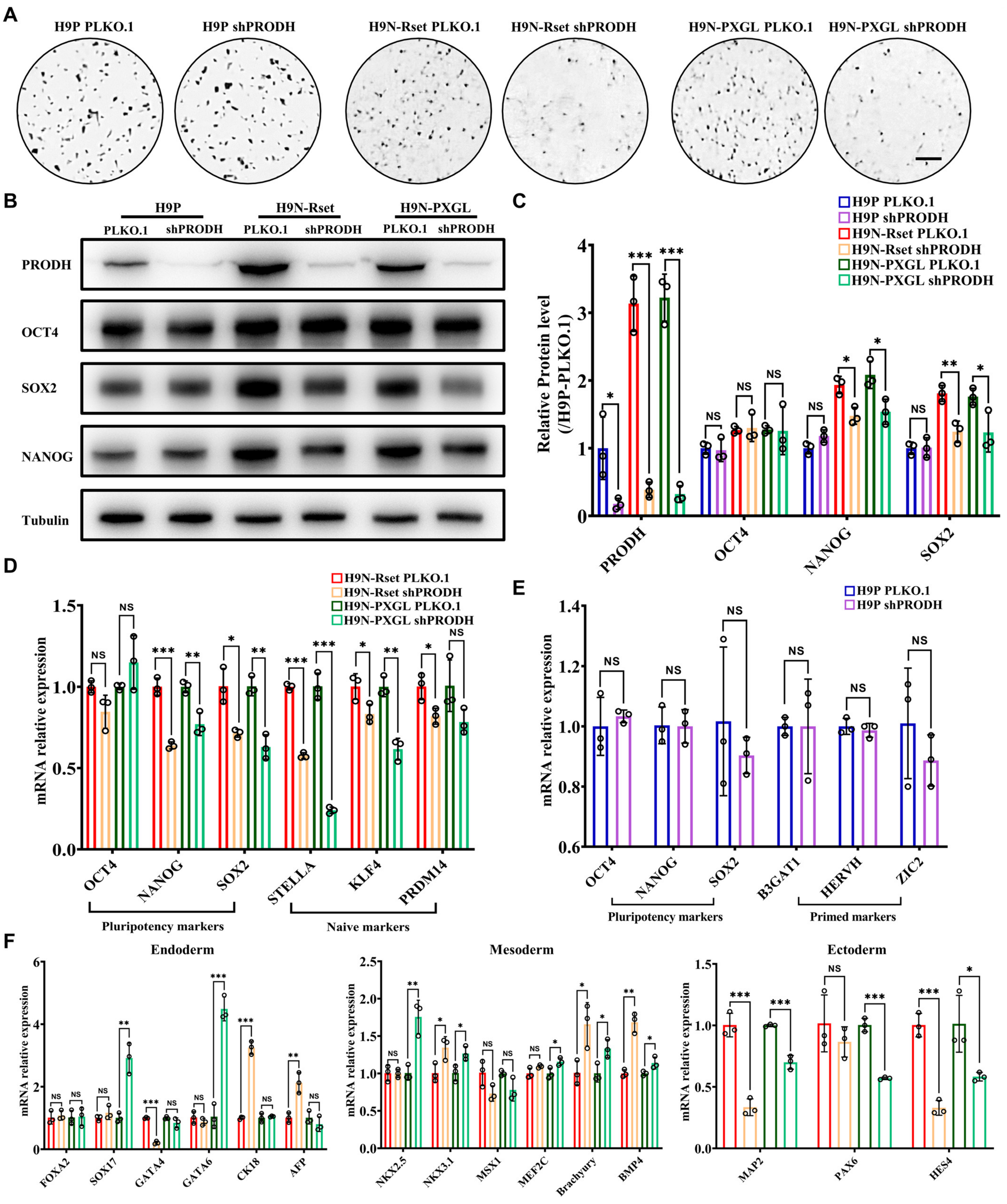
A
H9P PLKO.1
H9P shPRODH
H9N-Rset PLKO.1
H9N-Rset shPRODH
H9N-PXGL PLKO.1
H9N-PXGL shPRODH
B
H9P
H9N-Rset
H9N-PXGL
PLKO.1
shPRODH
PRODH
OCT4
SOX2
NANOG
Tubulin
C
Relative Protein level (/H9P-PLKO.1)
PRODH
OCT4
NANOG
SOX2
D
mRNA relative expression
H9N-Rset PLKO.1
H9N-Rset shPRODH
H9N-PXGL PLKO.1
H9N-PXGL shPRODH
OCT4
NANOG
SOX2
STELLA
KLF4
PRDM14
Pluripotency markers
Naive markers
E
H9P PLKO.1
H9P shPRODH
B3GAT1
HERVH
ZIC2
Primed markers
F
Endoderm
Mesoderm
Ectoderm
FOXA2
SOX17
GATA4
GATA6
CK18
AFP
NKX2.5
NKX3.1
MSX1
MEF2C
Brachyury
BMP4
MAP2
PAX6
HES4

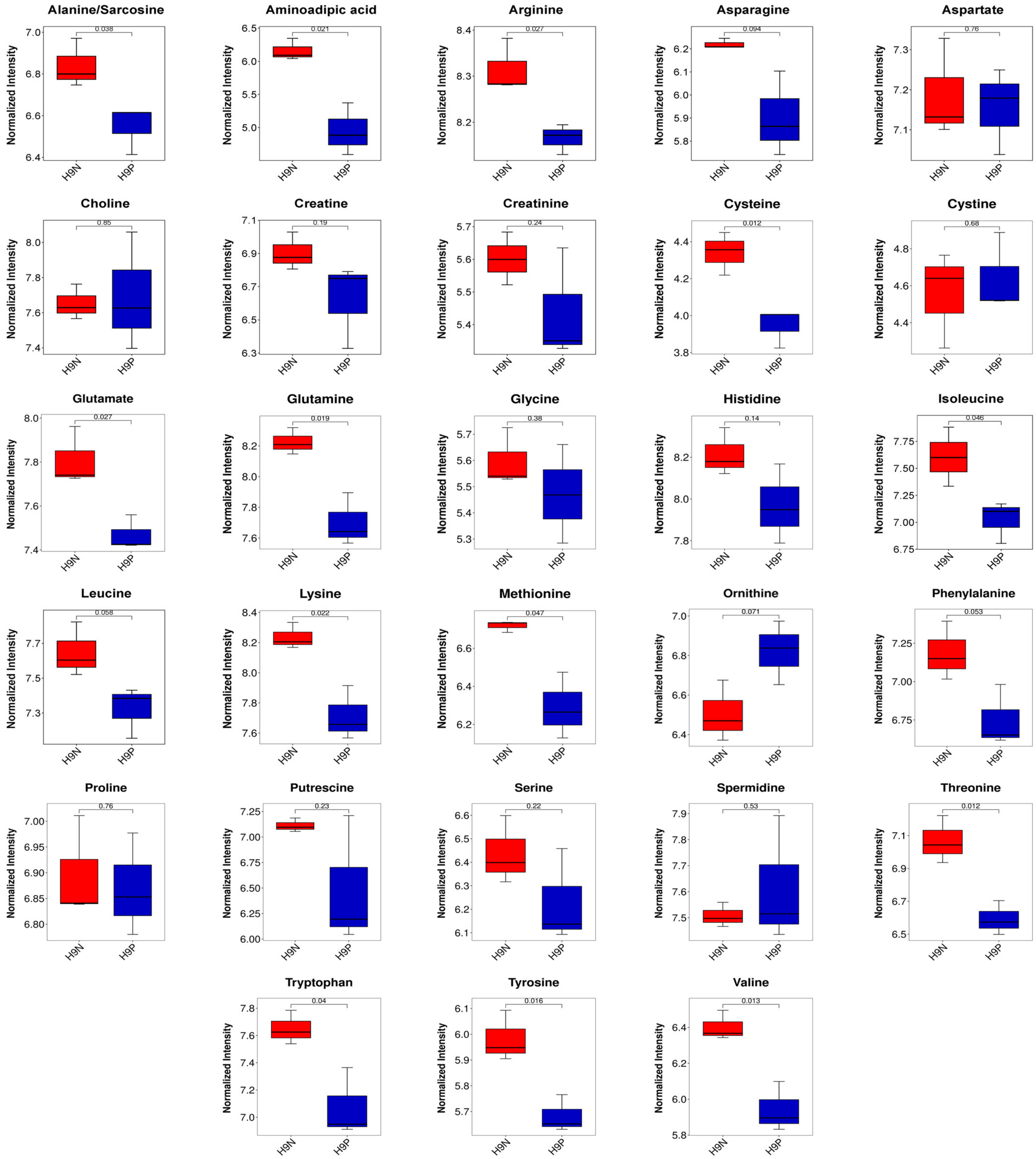

Figure EV2. Analysis of targeted metabolomics of amino acids and their derivatives between naive (Rset) and primed H9 hESCs.

The boxplots represent intensities of amino acids and their derivatives detected by targeted metabolomics. Normalized intensity, log10 (scale+1). Horizontal line represents median, box ranges represent values between quartiles 1 and 3, and whiskers represent values from minimum to maximum. Three biological replicates were analyzed. The statistical significance was analyzed using unpaired two-tailed Student's t-test.

 © The Author(s)

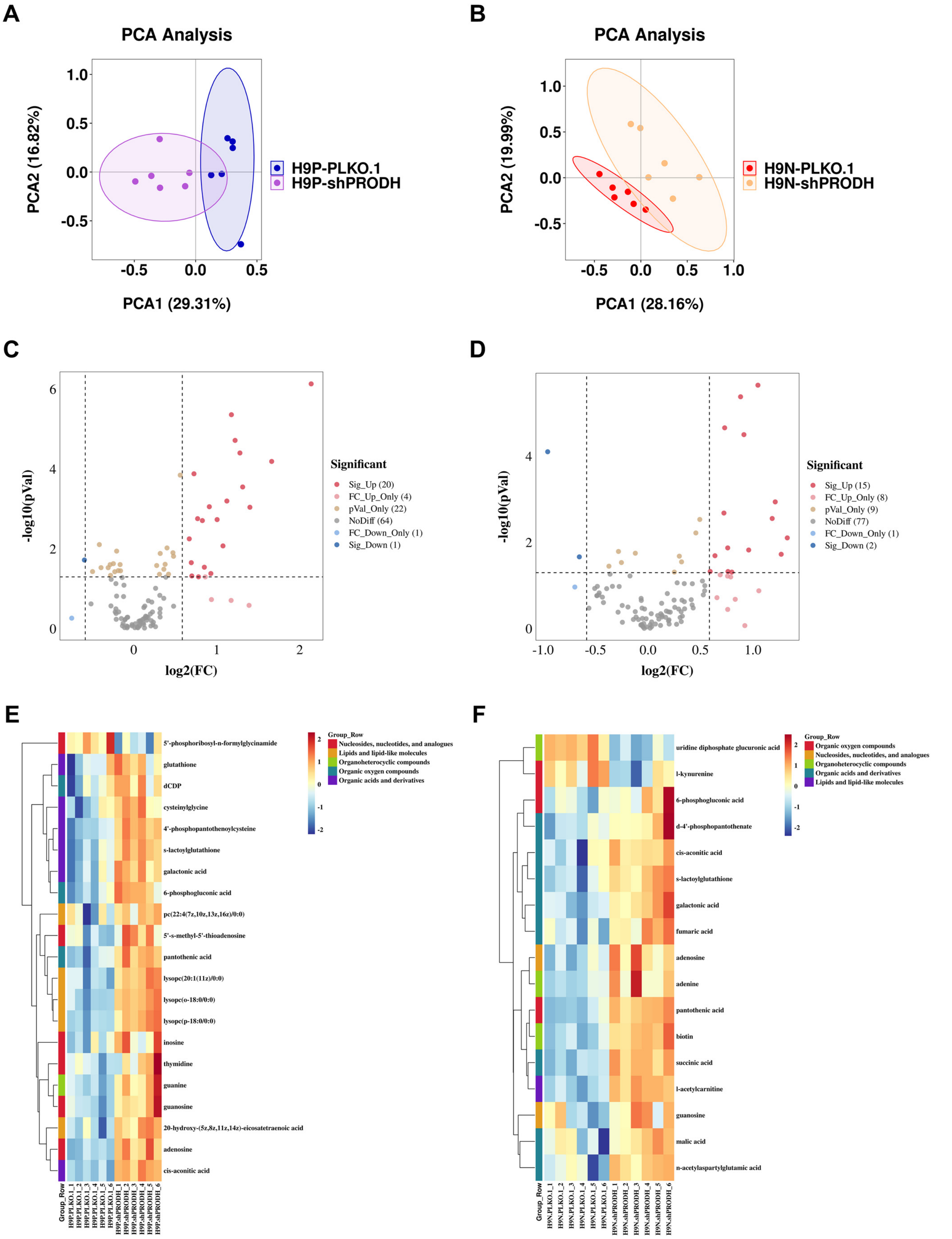
A
PCA Analysis
H9P-PLKO.1
H9P-shPRODH
PCA1 (29.31%)
PCA2 (16.82%)
B
PCA Analysis
H9N-PLKO.1
H9N-shPRODH
PCA1 (28.16%)
PCA2 (19.99%)
C
Significant
Sig_Up (20)
FC_Up_Only (4)
pVal_Only (22)
NoDiff (64)
FC_Down_Only (1)
Sig_Down (1)
log2(FC)
-log10(pVal)
D
Significant
Sig_Up (15)
FC_Up_Only (8)
pVal_Only (9)
NoDiff (77)
FC_Down_Only (1)
Sig_Down (2)
log2(FC)
-log10(pVal)
E
Group_Row
Nucleosides, nucleotides, and analogues
Lipids and lipid-like molecules
Organoheterocyclic compounds
Organic oxygen compounds
Organic acids and derivatives
5'-phosphoribosyl-n-formylglycinamide
glutathione
dCDP
cysteinylglycine
4'-phosphopantothenoylcysteine
s-lactoylglutathione
galactonic acid
6-phosphogluconic acid
pc(22:4(7z,10z,13z,16z)/0:0)
5'-s-methyl-5'-thioadenosine
pantothenic acid
lysopc(20:1(11z)/0:0)
lysopc(o-18:0/0:0)
lysopc(p-18:0/0:0)
inosine
thymidine
guanine
guanosine
20-hydroxy-(5z,8z,11z,14z)-eicosatetraenoic acid
adenosine
cis-aconitic acid
Group_Row
H9P.PLKO.1_1
H9P.PLKO.1_2
H9P.PLKO.1_3
H9P.PLKO.1_4
H9P.PLKO.1_5
H9P.PLKO.1_6
H9P.shPRODH_1
H9P.shPRODH_2
H9P.shPRODH_3
H9P.shPRODH_4
H9P.shPRODH_5
H9P.shPRODH_6
F
Group_Row
Organic oxygen compounds
Nucleosides, nucleotides, and analogues
Organoheterocyclic compounds
Organic acids and derivatives
Lipids and lipid-like molecules
uridine diphosphate glucuronic acid
l-kynurenine
6-phosphogluconic acid
d-4'-phosphopantothenate
cis-aconitic acid
s-lactoylglutathione
galactonic acid
fumaric acid
adenosine
adenine
pantothenic acid
biotin
succinic acid
l-acetylcarnitine
guanosine
malic acid
n-acetylaspartylglutamic acid
Group_Row
H9N.PLKO.1_1
H9N.PLKO.1_2
H9N.PLKO.1_3
H9N.PLKO.1_4
H9N.PLKO.1_5
H9N.PLKO.1_6
H9N.shPRODH_1
H9N.shPRODH_2
H9N.shPRODH_3
H9N.shPRODH_4
H9N.shPRODH_5
H9N.shPRODH_6

Figure EV3. The effect of PRODH knockdown on metabolism of primed versus naive (Rset) hESCs.

(**A**,**B**) Principal-component analyses for all identified metabolites in H9P-shPRODH versus H9P-PLKO.1 hESCs (**A**) and H9N-shPRODH versus H9N-PLKO.1 hESCs (**B**). (**C,D**) Distribution of significantly changed metabolites (P-value < 0.05, FC > 1.5 or <0.67, as indicated by the dashed lines) between H9P-shPRODH and H9P-PLKO.1 hESCs (**C**), as well as H9N-shPRODH and H9N-PLKO.1 hESCs (**D**). The statistical significance was analyzed using unpaired two-tailed Student's t-test. (**E**,**F**) Heatmap showing significantly altered metabolites between H9P-shPRODH and H9P-PLKO.1 hESCs (**E**), as well as H9N-shPRODH and H9N-PLKO.1 hESCs (**F**).

© The Author(s)

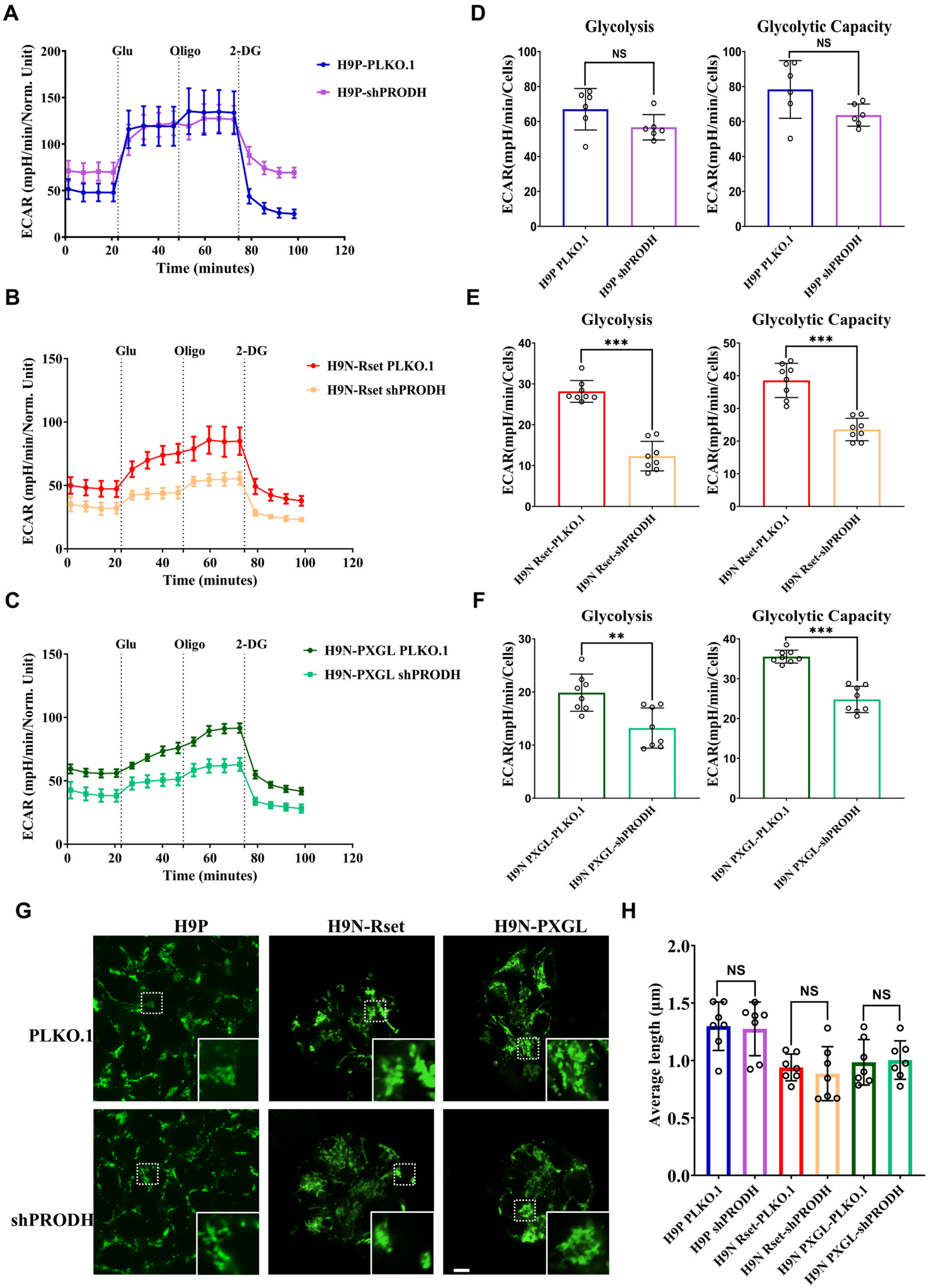
A
ECAR (mpH/min/Norm. Unit)
Glu
Oligo
2-DG
H9P-PLKO.1
H9P-shPRODH
Time (minutes)
B
H9N-Rset PLKO.1
H9N-Rset shPRODH
C
H9N-PXGL PLKO.1
H9N-PXGL shPRODH
D
Glycolysis
Glycolytic Capacity
NS
ECAR(mpH/min/Cells)
H9P PLKO.1
H9P shPRODH
E
***
H9N Rset-PLKO.1
H9N Rset-shPRODH
F
**
H9N PXGL-PLKO.1
H9N PXGL-shPRODH
G
H9P
H9N-Rset
H9N-PXGL
PLKO.1
shPRODH
H
Average length (μm)
H9P PLKO.1
H9P shPRODH
H9N Rset-PLKO.1
H9N Rset-shPRODH
H9N PXGL-PLKO.1
H9N PXGL-shPRODH

Figure EV4. Effect of PRODH knockdown on oxidative phosphorylation and glycolysis in primed versus naive hESCs.

(**A–C**) Glycolytic function of PLKO.1 and shPRODH hESCs was measured by ECAR, after sequential injections of glucose (Glu), oligomycin (Oligo), and 2-deoxyglucose (2-DG). Ten technical replicates were analyzed. (**D–F**) ECAR of PLKO.1 and shPRODH hESCs in mTeSR1 (**D**), Rset (**E**), and PXGL (**F**) mediums were detected and analyzed. Glycolysis was the increase in ECAR measured after the glucose injection. Glycolytic capacity was defined by subtracting ECAR with 2-DG from ECAR with oligomycin. (**G,H**) The length of mitochondria. Mitochondria were stained with MitoTracker Green for 30 min. Representative confocal micrographs are shown (**G**). Scale bars, 10 µm. Mitochondrial length were analyzed by the ImageJ software MiNA (**H**). Data information: The statistical significance was analyzed using unpaired two-tailed Student's t-test in panels. NS, not significant ($P > 0.05$). $^{**}P < 0.01$, $^{***}P < 0.001$. Data were presented as mean ± SD. In (**D–F**), each data point represents a technical replicate. In (**G** and **H**), data were obtained from seven independent experiments, and more than ten cells were analyzed in each experiment. Each data point represents the average mitochondrial length in each replicate.

 © The Author(s)

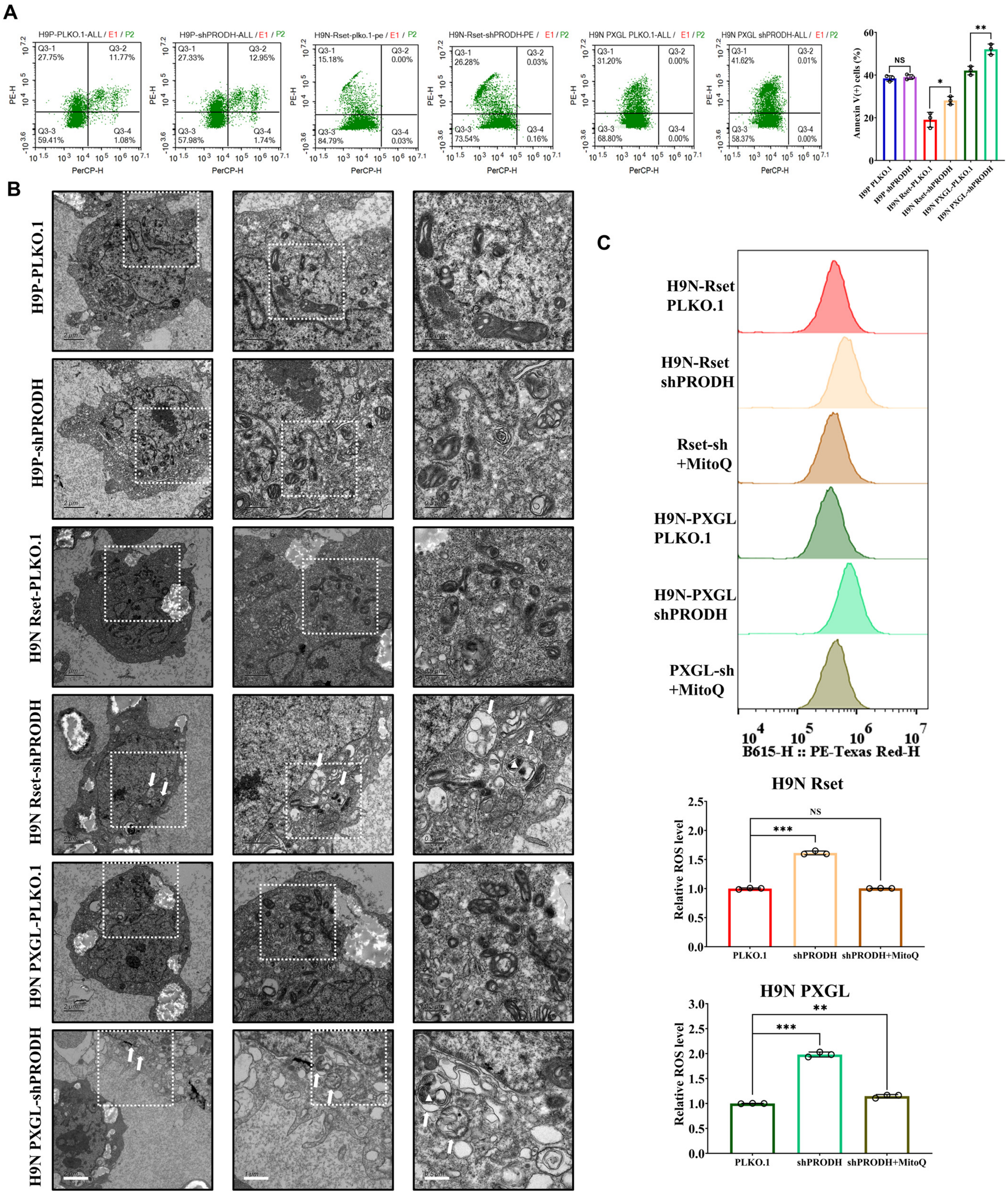
A
H9P-PLKO.1-ALL / E1 / P2
Q3-1 27.75%
Q3-2 11.77%
Q3-3 59.41%
Q3-4 1.08%
H9P-shPRODH-ALL / E1 / P2
Q3-1 27.33%
Q3-2 12.95%
Q3-3 57.98%
Q3-4 1.74%
H9N-Rset-plko.1-pe / E1 / P2
Q3-1 15.18%
Q3-2 0.00%
Q3-3 84.79%
Q3-4 0.03%
H9N-Rset-shPRODH-PE / E1 / P2
Q3-1 26.28%
Q3-2 0.03%
Q3-3 73.54%
Q3-4 0.16%
H9N PXGL PLKO.1-ALL / E1 / P2
Q3-1 31.20%
Q3-2 0.00%
Q3-3 68.80%
Q3-4 0.00%
H9N PXGL shPRODH-ALL / E1 / P2
Q3-1 41.62%
Q3-2 0.01%
Q3-3 58.37%
Q3-4 0.00%
PE-H
PerCP-H
Annexin V(+) cells (%)
NS
*
**
H9P PLKO.1
H9P shPRODH
H9N Rset-PLKO.1
H9N Rset-shPRODH
H9N PXGL-PLKO.1
H9N PXGL-shPRODH
B
H9P-PLKO.1
H9P-shPRODH
H9N Rset-PLKO.1
H9N Rset-shPRODH
H9N PXGL-PLKO.1
H9N PXGL-shPRODH
2 μm
1 μm
0.5 μm
C
H9N-Rset PLKO.1
H9N-Rset shPRODH
Rset-sh +MitoQ
H9N-PXGL PLKO.1
H9N-PXGL shPRODH
PXGL-sh +MitoQ
B615-H :: PE-Texas Red-H
H9N Rset
Relative ROS level
***
NS
PLKO.1
shPRODH
shPRODH+MitoQ
H9N PXGL
Relative ROS level
***
**
PLKO.1
shPRODH
shPRODH+MitoQ

Figure EV5. The impact of excessive ROS on naive hESCs.

(**A**) Cells stained with Annexin V and 7AAD were determined by flow cytometry. Live cells were defined as Annexin V-/7AAD-, early apoptosis AnnexinV + /7AAD-, and late apoptosis AnnexinV + /7AAD+. (**B**) Primed and naive (Rset and PXGL) hESCs were harvested for examination by transmission electron microscopy (TEM). The images were shown at various magnification levels. Scale bars, 2 µm (left), 1 µm (middle) 0.5 µm (right). The white arrows indicate AV-like organelles and the white triangles indicate mitochondria enclosed within AVs. (**C**) ROS levels were determined by MitoSOX. Rset-shPRODH and PXGL-shPRODH cells were treated with 50 nM MitoQ for 3 h, and cultured for an additional 12 h. Fluorescent intensity of cells stained with MitoSOX was determined by flow cytometry (upper), and quantitative data normalized to Control cell values (H9N Rset-PLKO.1 or H9N PXGL-PLKO.1) were presented (lower). Data information: The statistical significance was analyzed using unpaired two-tailed Student's t-test in panels. NS, not significant ($P > 0.05$). *$P < 0.05$, **$P < 0.01$, ***$P < 0.001$. The data shown were all from three independent biological replicates. Each data point represents an independent biological replicate. Data were presented as mean ± SD.

 © The Author(s)
